# Characterizing the microbial community constructure and the metabolites among different colour Moutai *Daqu*.

**DOI:** 10.1016/j.fochx.2025.102223

**Published:** 2025-01-25

**Authors:** Chao Chen, Derang Ni, Yubo Yang, Jinhu Tian, Fan Yang, Xingqian Ye

**Affiliations:** a Institute of Science and Technology, Kweichow Moutai Group, Renhuai, Zunyi, China; b College of Biosystems Engineering and Food Science, National-Local Joint Engineering Laboratory of Intelligent Food Technology and Equipment, Fuli Institute of Food Science, Zhejiang Key Laboratory for Agro-Food Processing, Zhejiang International Scientific and Technological Cooperation Base of Health Food Manufacturing and Quality Control, Zhejiang University, Hangzhou, China; c Zhejiang University Zhongyuan Institute, Zhengzhou, China

**Keywords:** *Daqu*, Baijiu, Microorganism, Liquefaction, Amino acid, Fatty acid

## Abstract

There are three types of *Daqu* produced during the fermentation of Moutai *Daqu*, which are named as white, yellow and black *Daqu*. However, in-depth studies for them are lacking. Herein, the high-throughput sequencing and metabolomics techniques were used to analyze the differences in Moutai *Daqu.* The findings indicated that the predominant microorganisms in yellow and white *Daqu* were *Kroppenstedtia* and *Bacillus*, while *Oceanbacillus* and *Scopulibacillus* emerged as the primary microorganisms in black *Daqu*. Further exploration revealed that white and black *Daqu* played important roles in the liquefaction and saccharification processes. Besides, the results of metabolomics reveals that yellow and black *Daqu* exhibit a higher abundance of up-regulated amino acids and fatty acids, which exert a more significant effect on Moutai Baijiu flavor and bioactivity. This study reveals the differences among the three types of Moutai *Daqu* through comprehensive analysis, which provides technical support for improving the quality of Moutai *Daqu*.

## Highlights

• Yellow *Daqu* had the lowest bacterial community richness and diversity among the three types of Moutai *Daqu*.
• *Kroppenstedtia* and *Bacillus* were characteristic genera of yellow and white Moutai *Daqu* respectively.
• *Oceanobacillus* and *Virgibacillus* were characteristic genera of black Moutai *Daqu.*
• Black and yellow *Daqu* contribute more to the flavor and functional activity of Moutai Baijiu.
• White *Daqu* contribute more to the yield of Moutai Baijiu.

## 1 Introduction

Baijiu is a kind of traditional Chinese brewing liquor, which divided into 12 flavor types. And sauce-flavor, strong-flavor and light-flavor are the 3 main flavor types. Sauce-flavor Baijiu is favored by consumers because of its mellow taste and long aroma, these characteristics are due to the production of many flavor compounds in the fermentation process (Li et al., 2022; Wang, Huang and Huang, 2021). The production process of sauce-flavor Baijiu is a traditional fermentation process handed down from ancient times, which belongs to the open brewing and called “12,987” for short. Its meaning mainly refers to the production cycle of 1 year, 2 feeding processes, 9 steaming, 8 fermentations and “7” is seven times of distillation. The raw materials of sauce-flavor Baijiu are wheat, sorghum and water. Wheat is fermented to form *Daqu* first, a saccharifying and fermenting agent. Sorghum is used as the substrate of *Daqu* during the 8 fermentations process, the steamed sorghum is mixed with *Daqu* at a ratio of 1:1. After the mixture is fully fermented, it is distilled to form the base Baijiu and blended by the master brewer.

*Daqu* plays a crucial role in the production of Baijiu by promoting substrate saccharification and fermentation. The shape of *Daqu* is like a brick, and it is formed by putting crushed wheat into the room and fermented after artificial stepping. *Daqu* is closely related to the flavor of sauce-flavor Baijiu (Yin et al., 2022). Previous research has demonstrated that *Daqu* contributed 9.1 % - 27.4 % of bacteria during the Baijiu fermentation process, which is an important source of brewing microorganisms (Wang, Du, Zhang, & Xu, 2018). In addition, *Daqu* contributed enzymes and metabolites for fermentation process (Sakandar, Hussain, Farid Khan, & Zhang, 2020).The enzymes could rapidly degrade the starch, protein and other macromolecules in the raw materials to form precursors such as sugars and amino acids, which are further fermented to form ethanol and other flavor metabolites (Tian et al., 2020). Therefore, the quality of *Daqu* is mainly related to the dominant microorganism, enzyme activity, and metabolites. The quality of *Daqu* determines whether the sauce-flavor Baijiu has a strong aroma and mellow taste (Zhang et al., 2022).

During the fermentation process of *Daqu*, approximately 80 % yellow *Daqu*, 10–15 % white *Daqu*, and around 1 % black *Daqu* are formed due to variations in the temperature and humidity of the fermentation room, inconsistent fermentation levels and other reasons (Cai et al., 2021). The proportion of the three types of *Daqu* used in production was determined according to the experience of the workers, and the effect of different types of *Daqu* on the yield and quality of Baijiu is still unknown. Clarifying the differences in microbial composition, enzyme activity and metabolite types among the three types of *Daqu* is beneficial to the quality control of Baijiu.

The flavor of *Daqu* used in the production of sauce-flavor Baijiu in different regions of China varies greatly. And the different microbial composition of *Daqu* produced in different regions may be one of the reasons for the unique flavor. For instance, Shi et al. found that the dominant microorganisms in yellow and black *Daqu* were *Kroppenstedtia*, and in white *Daqu* were *Oceanobacillus* and *Bacillus* (Shi et al., 2022). But in another study (Wang et al., 2021), they found that the three types of *Daqu* were dominated by *Thermoactinomyces*. Moreover, Deng et al. found that the white *Daqu* was dominated by *Kroppenstedtia*, *Bacillus* and *Lentibacillus*, and the yellow *Daqu* was dominated by *Kroppenstedtia* and *Staphylococcus*. Black *Daqu* was dominated by *Bacillus* and *Saccharopolyspora* (Deng et al., 2020). The *Daqu* used in these studies are all from different regions, indicating that the *Daqu* produced in a certain region should be targeted for research. As a representative of sauce-flavor Baijiu, Moutai is produced in Chishui river, Moutai Town, Zunyi City, Guizhou Province. Its core production area is only 15.03 km^2^, which has been proved to be a unique place to produce Moutai Baijiu. However, to the best of our knowledge, there have been some studies on the differences between the three types of *Daqu* in other sauce-flavor Baijiu, but few studies on the three types of Moutai *Daqu*, and the differences between them have not been reported.

Thus, high-throughput sequencing and metabolomics techniques were used to analyze the microbial diversity and metabolites of three types of Moutai *Daqu* in this study. The purpose was to clarify the differences among the three types of Moutai *Daqu* and to provide a theoretical basis for improving the quality of Moutai Baijiu.

## 2 Materials and methods

### 2.1 Sample collection

Three types of Moutai *Daqu* are provided from Kweichow Moutai Co., Ltd. The sample was formed by spontaneous fermentation of wheat in a room after manual trampling (Supplementary Fig. S1) and was cuboid in shape. Twenty seven *Daqu* bricks were randomly selected from 3 different warehouses, crushed, and mixed thoroughly, 9 samples (3 parallel samples each) of three types of *Daqu* were obtained from the selected bricks. The samples were then placed in sterile bags and stored at −80 °C until future use.

### 2.2 Analysis of enzyme activity

The activities of liquefaction, saccharification, fermentation and esterification enzymes were estimated according to the Light Industry Standard of the People's Republic of China (QB/T 4257–2011). Briefly, The activity of the liquefaction enzyme refers to the grams of starch that can be liquefied by one gram of *Daqu* per hour at 35 °C and pH 4.6 (U, g/g·h). Similarly, the activity of the saccharification enzyme refers to the milligrams of glucose that can be saccharified by one gram of *Daqu* at 35 °C and pH 4.6 (U, g/g·h). The activity of the fermentation enzyme refers to the grams of CO_2_ that is fermented from saccharide by 0.5 g of *Daqu* for 72 h at 30 °C (g/0.5 g·72 h). The activity of the esterification enzyme refers to the milligrams of ethyl hexanoate that is synthesized from hexanoic acid and ethyl alcohol by 50 g of *Daqu* for 7 days at 35 °C (mg/50 g·7d).

### 2.3 High-throughput sequencing analysis

DNA was extracted using the CTAB extraction kit (R30171-100 T, Shanghai yuanye Bio-Technology Co., Ltd). Following genomic DNA extraction, the purity and concentration of DNA were assessed via 1 % agarose gel electrophoresis. An appropriate quantity of sample DNA was withdrawn from the centrifuge tube and diluted with sterile water to a concentration of 1 ng/μL.

Primer-specific region: The 16S V3-V4 region is amplified using primers 515F and 806R. All PCR reactions were carried out with 15 μL of Phusion High-Fidelity PCR Master Mix (New England Biolabs), 0.2 μM of forward and reverse primers, and 10 ng template DNA. Thermal cycling consisted of initial denaturation at 98 °C for 1 min, followed by cycling at 98 °C (10 s), 50 °C (30 s), and 72 °C (30 s) for a total of 30 cycles, and elongation at 72 °C for 5 min. Subsequently, the PCR products were analyzed via electrophoresis on a 2 % agarose gel. Sequencing libraries were generated using NEB Next Ultra™ II FS DNA PCR-free Library Prep Kit (New England Biolabs), and the resulting library was checked with Qubit and real-time PCR. The following library qualification, sequencing was performed on the NovaSeq6000 platform using the PE250 lane.

Using Barcode sequences and PCR amplification primers, data obtained from offline machines are partitioned by sample. FLASH version 1.2.11 (http://ccb.jhu.edu/software/FLASH/) assembles reads per sample yielding Raw Tags; these undergo stringent filtering using Fastp software version 0.23.1 to yield high-quality Clean Tags data before comparison with Silva database(https://www.arb-silva.de/)for detection and removal of chimeric sequences producing effective tags (Edgar, Haas, Clemente, Quince, & Knight, 2011). The DADA2 module in QIIME2 (version QIIME2–202202) is then employed in Effective tags for noise reduction to generate an amplicon sequence variant table, using Silva138.1 database for taxonomic assignment.

### 2.4 Metabolite analysis of three types of *Daqu*

One hundred mg of *Daqu* sample was placed in an EP tube and mixed with 500 μL of 80 % methanol aqueous solution. The mixture was vortexed, allowed to stand at −5 °C for 5 min, and then centrifuged at 15000 RPM and 4 °C for 20 min. A certain amount of the supernatant was taken and diluted with LC - MS grade water to reduce the methanol content to 53 %. After centrifugation again at 15000 RPM and 4 °C for another 20 min, the resulting supernatant was injected into the LC-MS/MS system for analysis.

UHPLC-MS/MS analyses were performed using a chromatographic instrument (Vanquish UHPLC system, Thermo Fisher, Germany) coupled with a mass spectrometer (Orbitrap Q Exactive™ HF, Thermo Fisher, Germany) at Novo gene Co., Ltd. (Beijing, China). Samples were injected into a chromatographic column (Hypersil GOLD™ Peptide HPLC Column, 100 × 2.1 mm, 1.9 μm) using a 17-min linear gradient at a flow rate of 0.2 mL/min. The eluents for the positive polarity mode were eluent A (0.1 % FA in Water) and eluent B (Methanol). The eluents for the negative polarity mode were eluent A (5 mM ammoniumacetate, pH 9.0) and eluent B (Methanol).The solvent gradient was set as follows: 2 % B, 1.5 min; 2–85 % B, 3 min; 85–100 % B, 10 min; 100–2 % B, 10.1 min; 2 % B, 12 min. Mass spectrometer was operated in positive/negative polarity mode with a spray voltage of 3.5 kV, capillary temperature of 320 °C, sheath gas flow rate of 35 psi and aux gas flow rate of 10 L/min, S-lens RF level of 60, Aux gas heater temperature of 350 °C.

During data processing, the raw files are initially imported into CD 3.1 search software. For each metabolite, a basic screening was conducted based on parameters such as retention time and mass-to-charge ratio. A retention time tolerance of 0.2 min and a mass tolerance of 5 ppm were set to align peaks from different samples, thereby enhancing identification accuracy. Subsequently, peak extraction was carried out with a mass tolerance of 5 ppm, signal intensity tolerance of 30 %, signal-to-noise ratio of 3, minimum signal intensity, and summed ion information for quantitative analysis of peak areas. Concurrently, target ions are integrated and molecular formulas are predicted using molecular ion peaks and fragment ions. The results are compared with the mzCloud (https://www.mzcloud.org/), mzVault, and Masslist databases. Blank samples were utilized for background ion removal while original quantitative results were normalized using the formula: sample raw quantitative value (sample metabolite quantitative value total/QC1 sample metabolite quantitative value total), resulting in relative peak areas. Metabolites with a relative peak area CV exceeding 30 % in the QC samples were excluded before obtaining the final results for metabolite identification and relative quantification.

### 2.5 Statistical analysis

IBM SPSS Statistics 26.0 software was used for one-way analysis of variance (ANOVA) and Duncan's test to calculate significance between samples (*p* < 0.05). The Novogene cloud platform (https://magic.novogene.com) was used to deal with the original sequence and its online tools in species composition analysis, linear discriminant analysis (LEfSe), correlation analysis, and microbial function prediction; partial least squares-discriminant analysis (PLS-DA) was established using SIMCA-P 14 software to analyze the 3 types of *Daqu*. Origin 2022b was used for principal component analysis of three types of Moutai *Daqu* and for drawing of bar graphs.

## 3 Results and discussion

### 3.1 Diversity analysis of the different types of Moutai *Daqu*

**Fig. 1:**
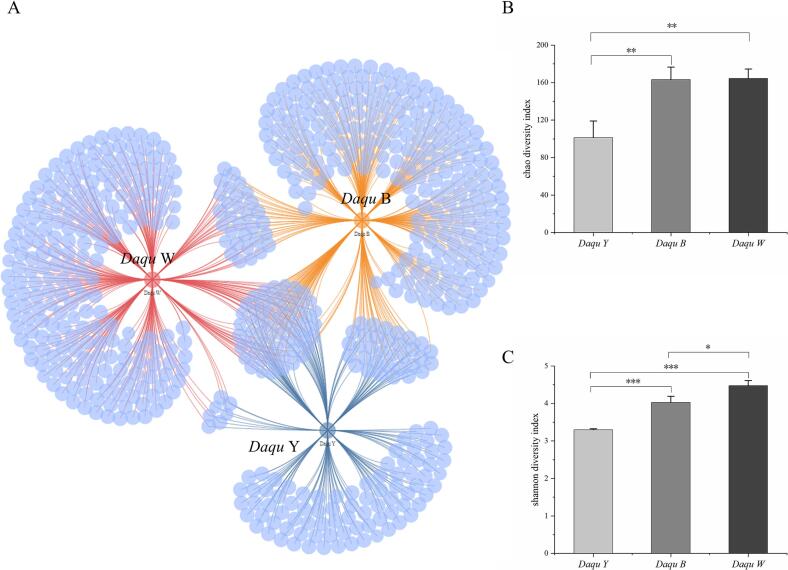
(A) Venn diagram of ASVs of three types of Moutai *Daqu*, (B) Chao diversity indexes of three types of Moutai *Daqu*, (C) Shannon diversity indexes of three types of Moutai *Daqu*.

High-throughput sequencing was performed to analyze the microbial diversity of three types of Moutai *Daqu*. The DADA2 method was used to process the raw data to obtain the de-repeated sequence ASV, which represents the abundance table of the sequences in the sample, which is called the feature table. In comparison with the traditional OUT method, ASVs enhanced the accuracy, comprehensiveness, and reproducibility of marker gene data analysis. Based on the obtained ASVs, we examined common and unique ASVs among the three types of Moutai *Daqu*. The Venn network analysis diagram in Fig. 1A illustrates the microbial composition at the ASV level for these samples. In total, 628 bacterial characteristic sequences were identified in all samples: black *Daqu* (329 species), yellow *Daqu* (179 species), and white *Daqu* (303 species). Notably, yellow *Daqu* exhibited significantly fewer characteristic sequences compared to black and white *Daqu*. Furthermore, there were 56 shared characteristic sequences across three types of *Daqu*, along with 83 unique characteristic sequences specific to yellow *Daqu* and 209 unique characteristic sequences specific to black and white *Daqu*. The results showed differences in the characteristic sequences of the three samples, with yellow *Daqu* having the fewest characteristic sequences.

In addition, Chao1 and Shannon indices were used to calculate bacterial α-diversity indices for the three samples. The Chao1 index, which is used to estimate the overall species richness of a given community sample, is positively correlated with species richness, The Shannon index was used to simultaneously characterize the diversity and evenness of species distribution within a sample of a given community. As shown in Fig. 1B, compared with yellow *Daqu*, the chao1 index of black and white *Daqu* was significantly increased by 64.32 % and 60.28 %, respectively (*p* < 0.05), while no significant difference between the chao1 index of black *Daqu* and white *Daqu* (*p* > 0.05). Similarly, as shown in Fig. 1C, compared with yellow *Daqu*, the Shannon index of black and white *Daqu* was significantly increased by 22.25 % and 35.75 %, respectively (*p* < 0.05). It is worth noting that yellow *Daqu* had the lowest richness and diversity at the bacterial level among the three types of *Daqu*. The reason for this phenomenon might attribute to the difference in temperature during the *Daqu* fermentation process. In fact, *Daqu* bricks were fermented in the room and placed in different locations. The temperature in the middle of the room is higher, and black *Daqu* is easily formed, while the temperature at the edge of the room is lower, and white *Daqu* is easily formed. This result is consistent with those of Shi et al., three types of *Daqu* produced by a sauce-flavor Baijiu production enterprise (Shi et al., 2022). They also found that yellow *Daqu* had the lowest bacterial community richness and diversity.

### 3.2 Comparative analysis of bacterial communities in three types of *Daqu*

**Fig. 2:**
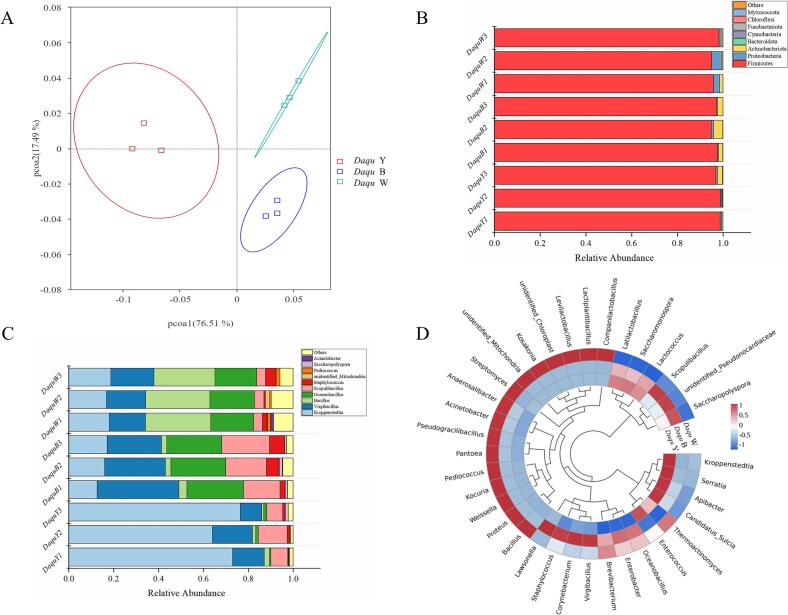
(A) PCA of the 3 types of *Daqu*, (B) Bar plots of bacterial communities of three types of Moutai *Daqu* at phylum level, (C) Bar plots of bacterial communities of three types of Moutai *Daqu* at genus levels, (D) Heatmap visualization of the top 35 genus in the three types of Moutai *Daqu*.

As shown in Fig. 2A, principal component analysis (PCA) was used to investigate the community structure of the three types of *Daqu*. The results showed that there was a considerable distance between the first and second principal components of the three types of *Daqu*, indicating significant differences in community structure. In contrast, samples from the same type of *Daqu* showed proximity, suggesting that the same type of *Daqu* had similar microbial compositions, and formed clusters. These results suggest that the formation of three types of *Daqu* during fermentation can be attributed to the different microbial community structures driving the fermentation.

As shown in Fig. 2B, At the phylum level, the three types of *Daqu* bacteria were predominantly from *Firmicutes*, with average relative abundances of 98.18 %, 96.55 %, and 96.33 % for three types of *Daqu* respectively. *Firmicutes* are highly tolerant to extreme environmental conditions and can grow normally under high temperatures and drought conditions (Chen, Li, Liu, & Lyu, 2024). The fermentation temperature of *Daqu* is usually higher than 60 °C, which would result in the failure of some poorly tolerant microorganisms to grow, thus making *Firmicutes* the dominant microorganism. This is consistent with the study by Zhang et al., who found that before fermentation, the dominant microorganism of *Daqu* was *Proteobacteria*, which gradually changed to *Firmicutes* with the progress of high temperature fermentation (Zhang et al., 2022). Therefore, high temperature fermentation might be the reason of *Firmicutes* were the dominant microorganism in *Daqu*.

The relative abundances of microorganisms at the genus level were further analyzed, and the results are shown in Fig. 2C. At the genus level, the dominant microorganism in yellow *Daqu* was *Kroppenstedtia*, accounting for 71.16 % of the total relative abundance. *Virgibacillus* and *Scopulibacillus* were dominant in black *Daqu*, accounting for 29.21 % and 18.55 % of the total relative abundance, respectively. White *Daqu* was dominated by *Bacillus* community, accounting for 28.28 % of the total relative abundance. In addition, *Oceanobacillus* accounted for only 1 % relative abundance in yellow *Daqu*, whereas it accounted for 24.69 % and 19.16 % relative abundance in black and white *Daqu*, respectively. From the community composition, it was found that black and white *Daqu* had similar community structures and complex community compositions. In the study by Shi et al., the relative abundance of the *Kroppenstedita* was the dominant microorganism in yellow *Daqu*, while *Bacillus* was the dominant microorganism in white *Daqu* (Shi et al., 2022). Similarly, Zhu et al.'s study revealed that the *Kroppenstedita* community was the dominant group in yellow *Daqu*, with an average relative abundance exceeding 66 % (Zhu et al., 2022). These research results were consistent with present results.

Furthermore, the top 35 genera were selected based on their abundance information in the three types of *Daqu* and clustered species at the genus level using a heatmap, as shown in Fig. 2D, it could be noticed that there were significant differences in the bacterial community distribution at the genus level among the three types of *Daqu*. The abundances of *Kroppenstedtia*, *Serratia*, *Apibacter*, and *Candidatus sulcia* in the yellow *Daqu* were significantly higher than other 2 samples. The relative abundance of *Virgibacillus*, *Corynebacterium*, *Staphylococcus*, *Lawsonnella*, *Scopulibacillus*, unidentified *Pseudonocardiaceae*, and *Saccharopolyspora* in black *Daqu* was significantly higher than other 2 samples. The abundance of *Bacillus*, *Proteus*, *Weissella* and 16 other genera in white *Daqu* was significantly higher than other 2 samples. This result was consistent with alpha-diversity index, which showed that low-abundance genus richness and community structure were higher and more complex in white *Daqu* compared than in yellow and black *Daqu*.

### 3.3 Characteristic bacterial genera in the three types of *Daqu*

**Fig. 3:**
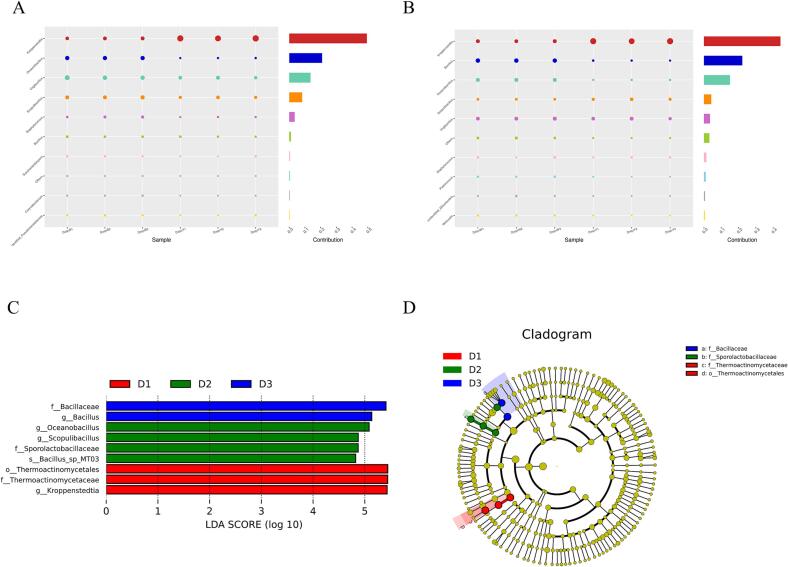
(A) Characteristic genus analysis of yellow and black Moutai *Daqu*, (B) Characteristic genus analysis of yellow and white Moutai *Daqu*, (C&D) Differences in the bacterial biomarkers among the three types of *Daqu*. D1: yellow *Daqu*, D2: black *Daqu*, D3: white *Daqu*. (For interpretation of the references to colour in this figure legend, the reader is referred to the web version of this article.)

In order to further understand the functional differences of the three samples, the characteristic bacterial genera or main microorganisms of the three samples and their roles in the brewing process of Baijiu were explored. In this study, Simper analysis was used to quantify the contribution of each species to the differences among the three samples. Lefse analysis was used to identify the main microorganism of the three samples. As shown in Fig. 3A and B, *Kroppenstedtia* was a characteristic genus of yellow *Daqu*; *Oceanobacillus* and *Virgibacillus* were characteristic genera of black *Daqu*; while *Bacillus* was a characteristic genus of white *Daqu*. Furthermore, Lefse analysis was used to identify biomarkers in the three samples. As shown in Fig. 3C and D, a total of 9 bacterial taxa exhibiting substantial differences in abundance (LDA threshold of 4) were identified across the three samples. At the genus level, *Kroppenstedtia* served as a bacterial marker for the yellow *Daqu*, while *Oceanbacillus* and *Scopulibacillus* functioned as the main microorganisms for the black *Daqu*, and *Bacillus* served as the main microorganism for the white *Daqu*. This result is consistent with the results of Simper analysis.

*Kroppenstedtia*, a thermophilic actinomycete widely reported in *Daqu*, is related to the synthesis of a variety of flavor compounds. It has been shown that *Kroppenstedtia* is a core genus involved in fatty acid biosynthesis during brewing and contributes to fatty acid production (Zhang et al., 2024). Fatty acids are important aroma and flavor components of sauce-flavor Baijiu, indicating that *Kroppenstedtia* is essential for flavor enhancement (Liu et al., 2024). Moreover, the abundance of *Kroppenstedtia* was positively correlated with the contents of 2,3,5-trimethylpyrazine, tetramethylpyrazine, hexal monomer, ethyl pentadecanoate, 2,4-dimethylbenzaldehyde and other flavor compounds (Wang, Huang, Hu, & Li, 2021). Therefore, the higher relative abundance of the *Kroppenstedtia* community in yellow *Daqu* positively impacts flavor and taste improvement.

Saccharifying enzymes are secreted by *Bacillus*, and the high relative abundance of *Bacillus* is conducive to the enhancement of *Daqu* saccharification power (He et al., 2019; Xu et al., 2022). Gan et al. observed a positive correlation between the average relative abundance of *Bacillus* and the saccharifying ability of *Daqu* (Gan et al., 2019). They identified α-glucosidase as the primary pathway for saccharification process, with *Bacillus* being the predominant microorganisms for α-glucosidase gene expression. In addition, many studies have demonstrated the ability of *Bacillus* to secrete large amounts of amylase and protease, which are essential for liquefaction and saccharification processes (Ding, Wu, Zhang, Zheng, & Zhou, 2014; Wang et al., 2014). Therefore, the relatively high abundance of *Bacillus* bacterial community might account for white *Daqu*'s elevated saccharifying ability. Furthermore, compared to other two types of *Daqu*, white *Daqu* exhibited greater diversity in community structure, higher protease activity, and lower acidity levels (Hou et al., 2024). Consequently, it displayed heightened fermentation activity among three types of *Daqu* and played a pivotal role in decomposing starch and protein substrates (Deng et al., 2020; Dong et al., 2024).

The dominant microorganisms in black *Daqu* were *Virgibacillus*, *Scopulibacillus* and *Oceanobacillus*, and their roles and functions in *Daqu* production and Baijiu brewing were not completely clear. It has been found that *Virgibacillus halodentriicans SK1–3-7* isolated from fish sauce fermentation had good protease secretion ability and could catalyze Tilapia hydrolysates with various biological activities (Montriwong, Kaewphuak, Rodtong, Roytrakul, & Yongsawatdigul, 2012). This suggested that *Virgibacillus* might affect the quality of *Daqu* through its protease activity and function. Moreover, Lv et al.studied the bacterial community succession and changes in volatile metabolites during shrimp paste fermentation (Lv et al., 2020). It was found that *Oceanobacillus* gradually became the predominant genus as fermentation progressed, and was positively correlated with aldehydes and pyrazines. Aldehydes and pyrazines are important flavor compounds in sauce-flavor Baijiu. Thus, the flavor of sauce-flavor Baijiu may be affected by *Oceanobacillus*.

### 3.4 Functional prediction of the three types of *Daqu*

**Fig. 4:**
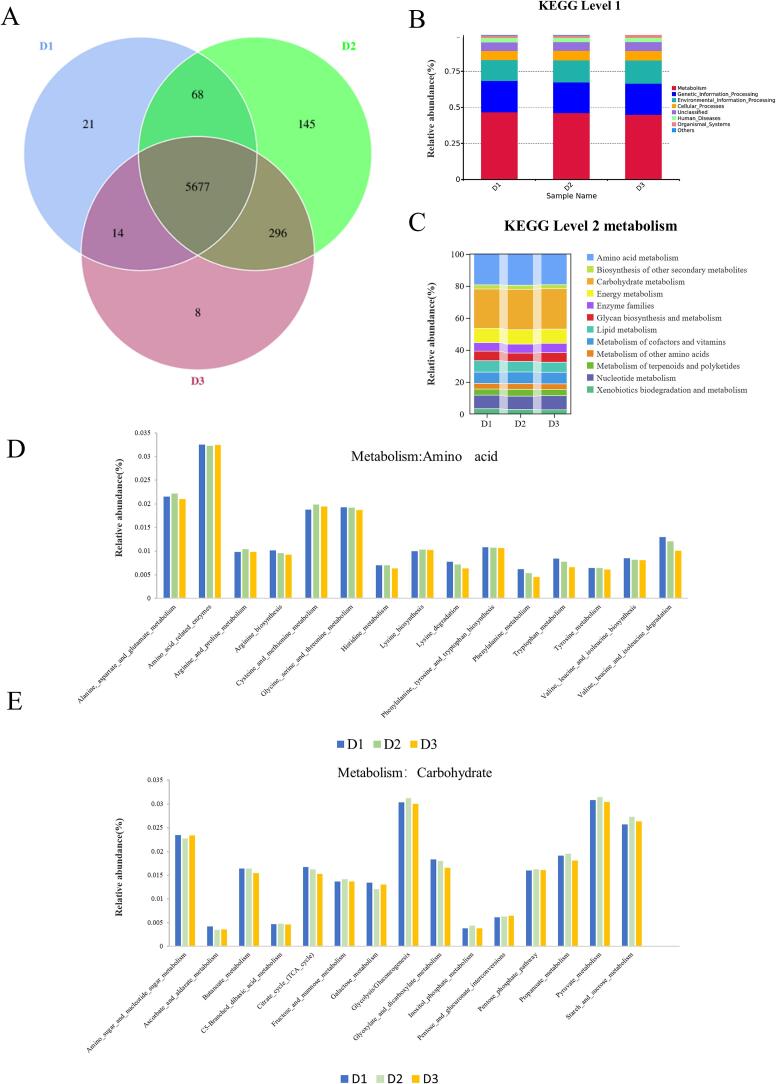
(A) Venn diagram of genes of three types of Moutai *Daqu*, (B) The functional annotation of Moutai *Daqu* microbiota genes by KEGG database at first level, (C) The functional annotation of Moutai *Daqu* microbiota genes by KEGG database at second level, (D&E) The functional annotation of Moutai *Daqu* microbiota genes by KEGG database at third level.

Further, PICRUSt2 was used to predict bacterial function and metabolism to better understand the role of the microbial communities in the three samples. As shown in Fig. 4A, the Venn diagram analysis was used to study the gene number distribution of the three samples, and 5677 common genes were identified, including 21 yellow *Daqu* genes, 145 black *Daqu* genes, and 8 white *Daqu* genes.

Subsequently, KEGG database was applied to annotate microbial community data and explore differences in functional features and metabolic pathways among the three types of *Daqu*. As shown in Fig. 4B, at the first level of the KEGG database, metabolism-related genes were found to be most abundant within microbial communities from all three types of *Daqu*, accounting for 46.75 %, 46.10 %, and 44.97 % respectively, within annotated genes from yellow, black, and white *Daqu* samples. These findings align with those reported by Huang et al., (Huang et al., 2024) who also observed that metabolism-related genes were predominant at this level within low-, medium-, and high-temperature *Daqu* groups with relative abundances of 59.99 %, 50.84 %, and 40.81 %, respectively.

In Baijiu fermentation, *Daqu* is mainly a catalyst for carbohydrate and amino acid metabolism. As shown in Fig. 4C, the relative abundance of amino acid metabolism-related genes in yellow, black, and white *Daqu* was 19.31 %, 19.52 %, and 19.21 %, respectively, while that of carbohydrate metabolism-related genes was 24.64 %, 25.28 %, and 25.28 %, respectively. These findings are consistent with Zhu et al.'s research outcomes (Zhu et al., 2023). As shown in Fig. 4D and E, the relative abundance of 30 metabolic pathways related to amino acid and carbohydrate metabolism in the three types of *Daqu* was also very similar. The above results indicate that three types of Moutai *Daqu* all have good abilities to process substrates.

### 3.5 Main functional analysis of the three types of *Daqu*

**Fig. 5:**
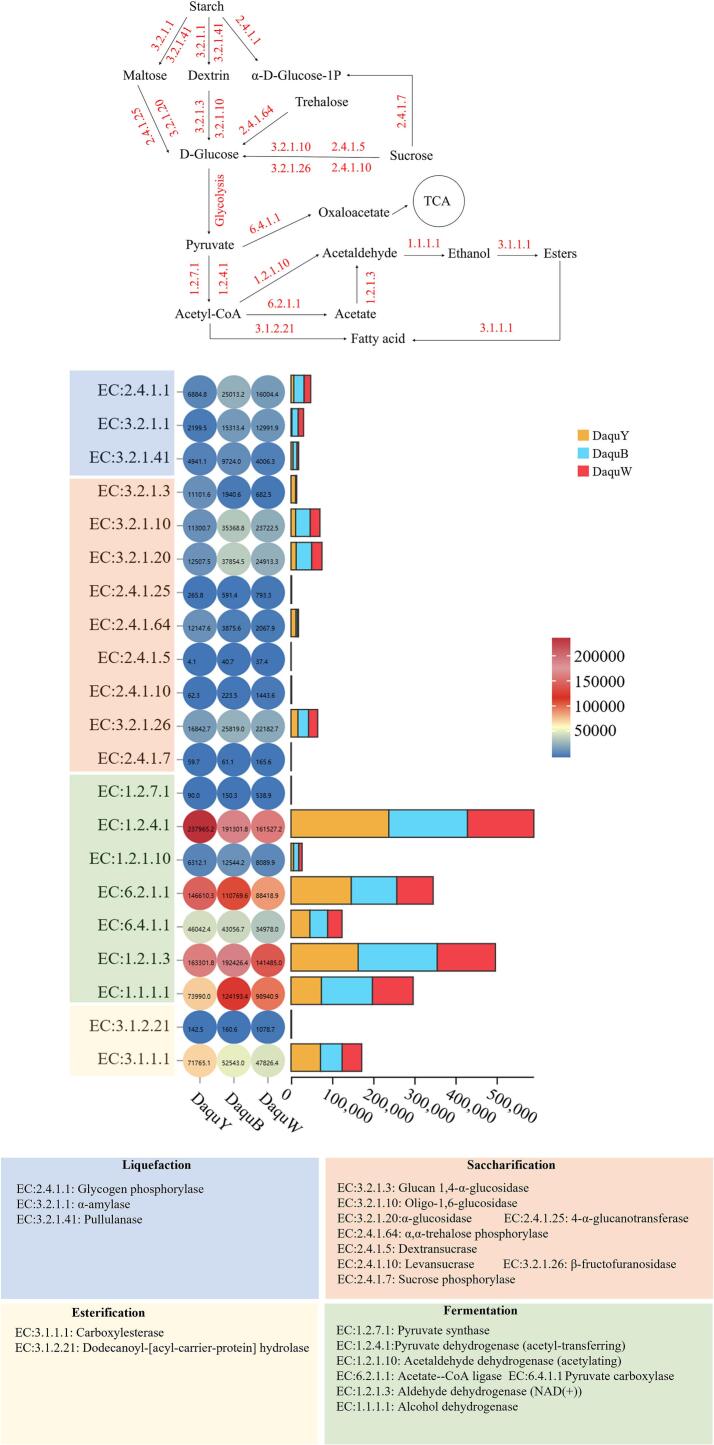
Analysis of predicted functional profiles for the three types of Moutai *Daqu*, heatmap of abundance of genes related to major functions of *Daqu*. (The value in the heatmap is the Transcripts Per Million (TPM) abundance of the enzyme, the abscissa of the stacked plot indicates the species grouping abundance).

During the brewing process, *Daqu* plays a crucial role in liquefaction, saccharification, fermentation, and esterification through the action of microorganism-secreted enzymes. Using the EC database, the absolute abundances of 21 genes associated with these four key functions of *Daqu* were assessed. As shown in Fig. 5, significant variations in the abundance of enzyme-coding genes related to *Daqu* were observed across different samples at the bacterial level.

The liquefaction power primarily refers to the enzymatic breakdown of large molecule starch granules into dextrin, maltose, and other components. The three enzymes involved in liquefaction were glycogen phosphorylase (EC:2.4.1.1), α-amylase (EC:3.2.1.1), and pullulanase (EC:3.2.1.41). Glycogen phosphorylase catalyzed the cleavage of the phosphoryl group from the 1,4-sugar glycosidic bond, releasing glucose residues to form α-d-glucose-1p. In contrast, α-amylase and pullulanase hydrolyze the α-1,4-sugar glycosidic bond in starch and the α-1,6-sugar glycosidic bond respectively, yielding dextrin and maltose. Among the three types of *Daqu*, the highest abundances of EC:2.4.1.1, EC:3.2.1.1 and EC:3.2.1.41 were found in black *Daqu*, while the abundance of EC:2.4.1.1 and EC:3.2.1.1 in white *Daqu* was also significantly higher than that in yellow *Daqu*, indicating that the liquefaction ability of black *Daqu* was the strongest and that of yellow *Daqu* was the weakest. In the study by Deng et al., the activity of starch metabolism-related enzymes in black *Daqu* was higher than that in yellow *Daqu* and white *Daqu*, which was consistent with the present study, suggesting that increasing the proportion of black *Daqu* in the brewing process was beneficial for starch liquefaction (Deng et al., 2020).

The saccharification power primarily involves the further breakdown of starch hydrolysis products, such as maltose, sucrose, and trehalose, into glucose. Enzymes including glucan 1,4-α-glucosidase (EC:3.2.1.3), oligo-1,6-glucosidase (EC:3.2.1.10), α-glucosidase (EC:3.2.1.20), 4-α-glucanotransferase (EC:2.4.1.25), and α, α-trehalose phosphorylase (EC:2.4.1.64) can catalyze the conversion of starch hydrolysis intermediates into glucose. Moreover, sucrose could undergo hydrolysis to yield glucose by the action of Dextransucrase (EC:2.4.1.5), oligo-1,6-glucosidase (EC:3.2.1.10), levansucrase (EC:2.4.1.10), and β-fructofuranosidase (EC:3.2.1.26). The relative abundances of EC:3.2.1.10, EC:3.2.1.20, EC:2.4.1.25, EC:2.4.1.5, EC:2.4.1.10, and EC:3.2.1.26 in yellow *Daqu* were comparatively lower than those in black and white *Daqu*. The aforementioned enzymes primarily participate in the hydrolysis of starch, maltose, and sucrose into glucose, which may account for the higher saccharifying potential observed in black and white *Daqu* compared to yellow *Daqu*.

The researchers investigated (Deng et al., 2020; Ge et al., 2024) the three types of *Daqu* (yellow, white, and black), and noted that both white and black *Daqu* exhibited stronger saccharifying capabilities than yellow *Daqu*. Concurrently, the relative abundance of genes associated with saccharification enzymes in black and white *Daqu* was significantly greater than that found in yellow *Daqu*, aligning with the findings of present study. In their study (Deng et al., 2020; Ge et al., 2024), a variety of *Daqu* from different production areas and manufacturers in China was utilized, demonstrating the consistent saccharification ability of the three types of *Daqu*. When necessary to enhance substrate saccharification during production, the proportion of black and white *Daqu* should be increased.

The fermentation power primarily refers to the capacity of *Daqu* microorganisms to metabolize glucose into ethanol via the pyruvate metabolism pathway. Enzymes such as pyruvate synthase (EC:1.2.7.1), pyruvate dehydrogenase (acetyl-transferring) (EC:1.2.4.1), and pyruvate carboxylase (EC6.4.1.1) facilitate the conversion of pyruvate into acetyl-CoA and oxaloacetate, which further undergoes metabolic processes leading to acetate, acetaldehyde, and fatty acid via the catalysis of enzymes such as acetaldehyde dehydrogenase (acetylating) (EC:1.2.1.10), acetate—CoA ligase (EC:6.2.1.1), and aldehyde dehydrogenase (Nad(+)) (EC:1.2.1.3). Ultimately, alcohol dehydrogenase (EC1.1.1.1) participates in this metabolic process by converting acetaldehyde into ethanol. As shown in the Fig. 5, the abundance of enzymes associated with fermentation (EC:1.2.4.1, EC:6.2.1.1, EC:1.2.1.3, EC:1.1.1.1) was significantly higher than that of other function-related enzymes, suggesting robust fermentation capacity in the *Daqu* samples. With the exception of a lower abundance of EC:1.2.7.11 in yellow *Daqu*, similar levels of other fermentation-related enzyme genes indicated comparable bacterial-level fermentation capacities across all three *Daqu* samples. In Mu et al.'s study, the enrichment of *Bacillus* and *Kroppenstedtia* genera was found to enhance the activity of fermentation-related enzymes and subsequently improve *Daqu*'s fermentation capacity; these two genera exhibit high relative abundance across all three *Daqu* samples and might be pivotal bacterial genera contributing to enhanced fermentation capacity (Mu et al., 2023).

The esterification power mainly refers to *Daqu*'s ability to synthesize the corresponding esters from alcohols and acids. The enzymes involved in ester synthesis are mainly Carboxylesterase (EC:3.1.1.1) and Dodecanoyl-[acyl-carrier-protein] hydrolase (EC:3.1.2.21), which can convert fatty acids and ethanol into esters. The relative abundance of EC3.1.1.1 in yellow *Daqu* is higher than that in black and white *Daqu*, but the relative abundance of EC3.1.2.21 is lower than that in black and white *Daqu*.

In order to verify the prediction results of ECs, the liquefaction, saccharification, fermentation, and esterification powers of the three types of *Daqu* were further evaluated. As shown in Table S1, the liquefaction and saccharification powers of both white *Daqu* and black *Daqu* are higher than those of yellow *Daqu*, which were consistent with the predicted results. The fermentation powers of yellow *Daqu* and white *Daqu* are close, and black *Daqu* is the highest. The esterification powers of white *Daqu* and black *Daqu* are close, while the esterification power of yellow *Daqu* is the lowest. These results indirectly reflect the microbial abundance in different *Daqu.*

### 3.6 PCA and PLS-DA of the three types of *Daqu* metabolites

QC samples are used to balance the chromatography-mass spectrometry system and instrument status to obtain accurate metabolomics data. As shown in Fig. S2, in positive ion mode and negative ion mode, both the ion peak baseline of three parallel QC samples is stable, and the peak pattern is consistent, indicating that the stability of the data was in high quality.

**Fig. 6:**
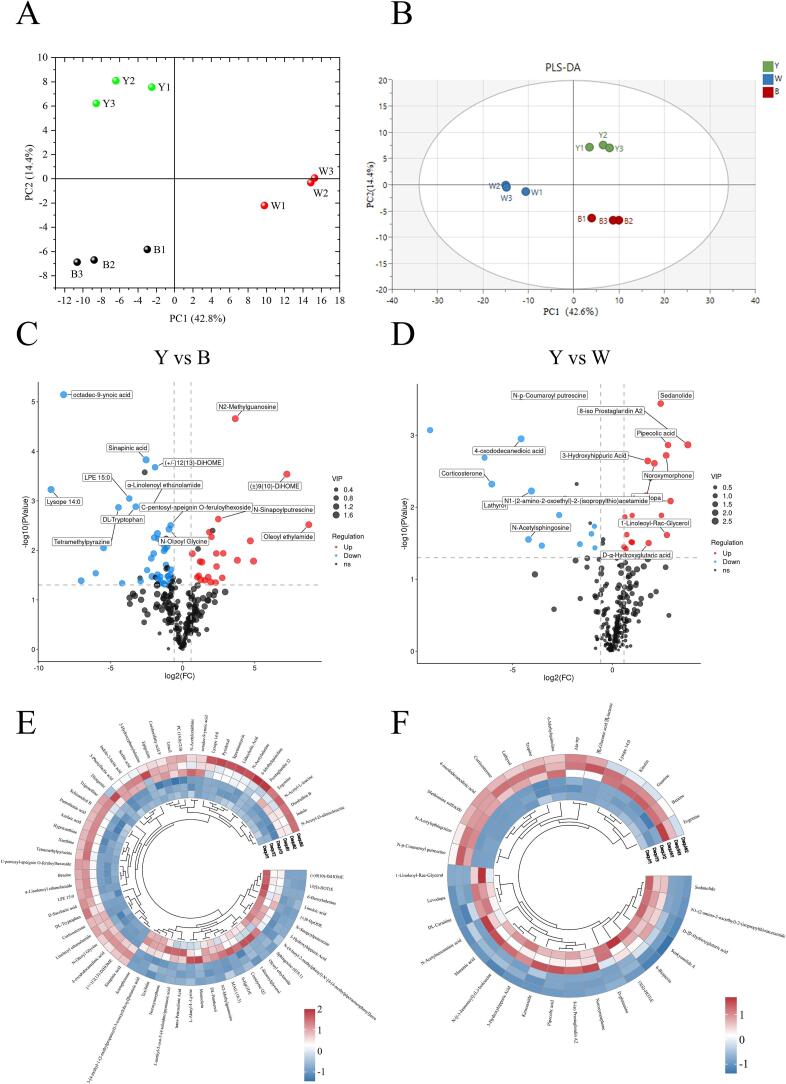
(A&B) PCA and PLS-DA of the three types of Moutai *Daqu* metabolites, (C) Volcano plot of differential metabolites in yellow and black Moutai *Daqu*, (D) Volcano plot of differential metabolites yellow and white Moutai *Daqu*, (E) Heatmap analysis of differential metabolites yellow and black Moutai *Daqu*, (F) Heatmap analysis of differential metabolites in yellow and white Moutai *Daqu*, Y: yellow *Daqu*, B: black *Daqu*, W: white *Daqu*. (For interpretation of the references to colour in this figure legend, the reader is referred to the web version of this article.)

Considering that microbial differences could lead to differences among the metabolites of the three samples, the analysis of non-volatile metabolites helps to further understand the functional differences among the three samples. The names, retention times, and relative quantitative values of all metabolites detected in the samples are listed in Supplementary Table S2. A total of 253 metabolites with different relative abundances were identified in the three samples. As shown in Fig. 6A, the PCA of the three samples could be separated into first and second principal components, indicating significant metabolite differences among the different types of *Daqu*. In addition, there were few metabolite differences within the same type of *Daqu*, indicating that the metabolite composition structure was similar.

Compared with PCA, PLS-DA is a supervised discriminant analysis method that enables a more precise analysis of group differences. As shown in Fig. 6B, the results of the PLS-DA revealed substantial disparities in metabolites among the three types of *Daqu*. The inter-group variations in metabolites were subtle, consistent with the PCA fingdings. Our findings align with those of Luo et al., who conducted an extensive examination on yellow, black, and white *Daqu* and identified notable metabolic differences across three types of *Daqu* (Luo et al., 2022).

### 3.7 Screening and volcano plotting of three types of *Daqu* metabolites

Fig. 6C and D show the detection of a total of 66 differential metabolites between yellow and black *Daqu*, as well as 33 differential metabolites between yellow and white *Daqu* (*p* < 0.05, VIP > 1, FC > 1.5 or FC > 1.2 or FC < 0.833; Supplementary Table S3 and S4). The upregulated metabolites of yellow *Daqu* included 3 Benzenoids, 13 Lipids and lipid-like molecules, 1 Phenylpropanoids and polyketides, 4 Organic acids and derivatives, 3 Organic oxygen compounds, 3 Organoheterocyclic compounds, and 1 Phenylpropanoids and polyketides. The upregulated metabolites of black *Daqu* included 2 Alkaloids and derivatives, 11 Lipids and lipid-like molecules, 7 Organic acids and derivatives, 11 Organoheterocyclic compounds, 4 Phenylpropanoids and polyketides, 2 Organic nitrogen compounds, along with 2 Organic oxygen compounds. The upregulated metabolites of black *Daqu* included 2 Alkaloids and derivatives, 4 Lipidsand lipid-like molecules, 3 Organic acids and derivatives, 3 Organoheterocyclic compounds, 1 Phenylpropanoids and polyketides. Notably, the differences between yellow and black *Daqu* were greater than those between yellow and white *Daqu*, with more differential metabolites.

### 3.8 Differential analysis of amino acids, their derivatives and fatty acids in different types of *Daqu*

Metabolites in *Daqu* are precursors of many flavor compounds in Baijiu and significantly contribute to the quality of Baijiu. The difference in the composition of metabolites in *Daqu* will have a great influence on the flavor of Baijiu. As shown in Fig. 6E and F, the differential metabolites among the three samples were shown in the heatmap. Among them, there were a variety of amino acids and derivatives that were significantly different. Amino acids are important taste compounds in the nonvolatile components of sauce-flavor Baijiu (Procopio, Sprung, & Becker, 2015), which have various biological activities (Niu et al., 2023). In addition, *Daqu* is fermented at high temperatures, which accelerates the breakdown of proteins and amino acids and promotes the Maillard reaction to produce important flavor components such as furfuryl alcohol, pyrazine, and aromatic compounds (Wang, Yang, Zhao, Zhang and Su, 2019). Meanwhile, amino acids are used as substrates by lactic acid bacteria and yeast in *Daqu*, thereby enhancing their esterase activity to produce more ester-flavored compounds (Chen et al., 2022). Therefore, amino acids and derivatives are important metabolites that affect the quality of *Daqu*.

Further analysis revealed that the relative total content was similar in the three samples (Supplementary Fig. S3), but there were differences in the upregulated amino acids and derivatives. To be specific, 41 amino acids and derivatives were detected in the three types of *Daqu*. There were significant differences in the content, with 4 upregulated amino acids and derivatives in yellow *Daqu*, 7 in black *Daqu*, and 3 in white *Daqu*. As shown in Fig. 6E and F, the upregulated amino acids and derivatives in yellow *Daqu* were *L*-Alanyl-l-Lysine, *N*-[(−)-Jasmonoyl]-(*L*)-Isoleucine, pipecolic acid, and *d*-glutamine. The upregulated amino acids and derivatives in black *Daqu* are *N*-Acetyl-*D*-alloisoleucine, *N*-Acetylalanine, *N*-Acetylornithine, *N*-Acetyl-*L*-leucine, Betaine, *N*-Oleoyl Glycine, and 2-Hydroxyphenylalanine. The upregulated amino acids and derivatives in white *Daqu* are Betaine, Methionine sulfoxide, and Ala-trp.

Among the upregulated amino acids and derivatives in yellow *Daqu*, *L*-Alanyl-*l*-Lysine is fermented to produce 2,3-dimethylpyrazine, 2-ethyl-3,6-dimethylpyrazine, and other pyrazine-flavored compounds, which are important flavor compounds in sauce-flavor Baijiu. In addition, pipecolic acid upregulated by yellow *Daqu* was found to inhibit fat accumulation and improve diabetes, which may have an impact on the functional activity of Baijiu (Luo et al., 2023). Among the upregulated amino acids and derivatives in black *Daqu*, *N*-Acetyl-L-leucine was found to improve neural function and was used to treat lysosomal storage disorders (Tifft Cynthia, 2024). These results suggest that the different microbial compositions could cause the formation of different amino acid metabolites in the three samples, which contributed to the Baijiu quality in different ways.

In addition to amino acids and derivatives, there were more differential lipid metabolites in the three samples. Lipids are esters and their derivatives are formed by fatty acids and alcohols, which can form a variety of products through oxidation and hydrolysis and are sources of flavor compounds in sauce-flavor Baijiu (Zou, Mu, Qi, Ren, & Su, 2024). In total 65 lipid substances were identified in *Daqu*, including 26 fatty acid compounds. The relative total content of the fatty acids in three types of *Daqu* exhibit similarity (Supplementary Fig. S4). Yellow *Daqu* contained 4 upregulated lipid compounds, black *Daqu* contained 11, and white *Daqu* contained 4. Specifically, the fatty acid compounds found in yellow *Daqu* included trans-Petroselinic acid, DL-Panthenol, and oleoyl ethylamide; those found in black *Daqu* included (+/−)12(13)-DiHOME, azelaic acid, and sorbic acid; while none were detected in white *Daqu*. The metabolic pathways of these fatty acids in *Daqu* and their contribution to flavor are unknown. Esters are important flavor compounds in Chinese Baijiu, which are beneficial to health. A total of 508 different microorganisms have been identified to influence ester synthesis through amino acid and fatty acid metabolism. However, few studies have investigated fatty acid metabolic pathways. Research has shown that understanding the catalytic mechanism of acetyltransferase/thiolase is of great significance for studying the metabolism and synthesis of fatty acids during Baijiu fermentation (Xu et al., 2022).

### 3.9 Correlation analysis of dominant genera and metabolites

**Fig. 7:**
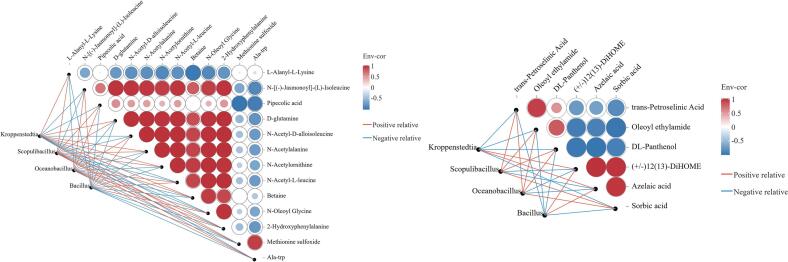
Correlation network plots between biomarkers, fatty acid, amino acid and derivatives of three types of Moutai *Daqu*. The red and blue lines indicate positive (*r* > 0.7, *p* < 0.05) and negative correlations (*r* < − 0.7, *p* < 0.05), respectively. (For interpretation of the references to colour in this figure legend, the reader is referred to the web version of this article.)

The metabolites in *Daqu* are produced by the fermentation of substrates such as proteins and carbohydrates by microorganisms. Therefore, biomarkers in three types of *Daqu*, differential metabolites of amino acids, their derivatives and fatty acids were processed for correlation analysis. Pearson correlation coefficients (|r| > 0.7, *p* < 0.05) were applied to network heat map analysis. As shown in Fig. 7, the biomarker genus *Kroppenstedtia* in yellow *Daqu* was positively correlated with 2 fatty acids, 6 amino acids and derivatives, negatively correlated with 4 fatty acids, 7 amino acids and derivatives. The biomarker genus *Scopulibacillus* in black *Daqu* was positively correlated with 4 fatty acids, 9 amino acids and derivatives, negatively correlated with 2 fatty acids, 4 amino acids and derivatives. *Oceanbacillus* was positively correlated with 4 fatty acids, 7 amino acids and derivatives, negatively correlated with 2 fatty acids, 6 amino acids and derivatives. *Bacillus* in white *Daqu* was positively correlated with 2 fatty acids, 4 amino acids and derivatives, negatively correlated with 4 fatty acids, 9 amino acids and derivatives. Among the amino acids and derivatives, *L*-alanyl-*l*-Lysine, Methionine sulfoxide, and Ala-trp were negatively correlated with the other 11 amino acids and derivatives, whereas all other amino acids and derivatives were positively correlated with each other. The results showed that the dominant bacterial genus only promoted the metabolism of certain fatty acids, amino acids and derivatives. The bacterial genera *Scopulibacillus* and *Oceanbacillus* found in black *Daqu* were positively correlated with the metabolism of more amino acids, their derivatives and fatty acids, which might explain why more lipids, amino acids and derivatives are upregulated in black *Daqu* compared to yellow and white *Daqu*.

## 4 Conclusion

To this day, our understanding of the three types of Moutai *Daqu* is still limited. In the present study, three types of Moutai *Daqu* were investigated using high-throughput sequencing and metabolomics techniques to clarify their functional differences. Results showed that the microbial composition, main microorganisms, important functions (liquefaction, saccharification), and metabolites of the three types of Moutai *Daqu* are significantly different, and the three samples have different contributions to the flavor and quality of Moutai Baijiu. Briefly, yellow and black *Daqu* might contribute more to the flavor and functional activity of Moutai Baijiu. White *Daqu* may contribute more to the yield of Moutai Baijiu because of its higher liquefaction and saccharification powers. The results of the present study provide valuable data on the diversity of the different types of *Daqu* in Moutai Baijiu production and provide ideas for optimizing the production process.

## CRediT authorship contribution statement

**Chao Chen:** Writing – original draft, Visualization, Investigation, Formal analysis. **Derang Ni:** Writing – review & editing, Methodology, Investigation. **Yubo Yang:** Supervision, Methodology, Data curation. **Jinhu Tian:** Writing – review & editing, Resources. **Fan Yang:** Validation, Resources, Project administration. **Xingqian Ye:** Writing – review & editing, Resources, Project administration, Funding acquisition, Conceptualization.

## Declaration of competing interest

The authors declare that they have no known competing financial interests or personal relationships that could have appeared to influence the work reported in this paper.

## Data Availability

The authors do not have permission to share data.
