# Supplementary material for: Characterizing the microbial community constructure and the metabolites among different colour Moutai Daqu
Source: Food Chem X. 2025 Jan 25;26:102223. doi: 10.1016/j.fochx.2025.102223 (PMC11872403; doi:10.1016/j.fochx.2025.102223)
Supplement: Supplementary material [file mmc1.docx]

**Supplementary Materials**

**Characterizing the microbial community constructure and the metabolites among different color Moutai *Daqu*.**

Chao Chen^a^, Derang Ni^a,b^, Yubo Yang^a^, Jinhu Tian^b^, Fan Yang^a,*^, Xingqian Ye^b,c*^

^a^ Institute of Science and Technology, Kweichow Moutai Group, Renhuai, Zunyi, China

^b^ College of Biosystems Engineering and Food Science, National-Local Joint Engineering Laboratory of Intelligent Food Technology and Equipment, Fuli Institute of Food Science, Zhejiang Key Laboratory for Agro-Food Processing, Zhejiang International Scientific and Technological Cooperation Base of Health Food Manufacturing and Quality Control, Zhejiang University, Hangzhou, China

c Zhejiang University Zhongyuan Institute, Zhengzhou, China

*Corresponding author:

Email: psu@zju.edu.cn (Ye XQ) ; yangfanmt@189.cn

Cell: +86(0571-88912162)


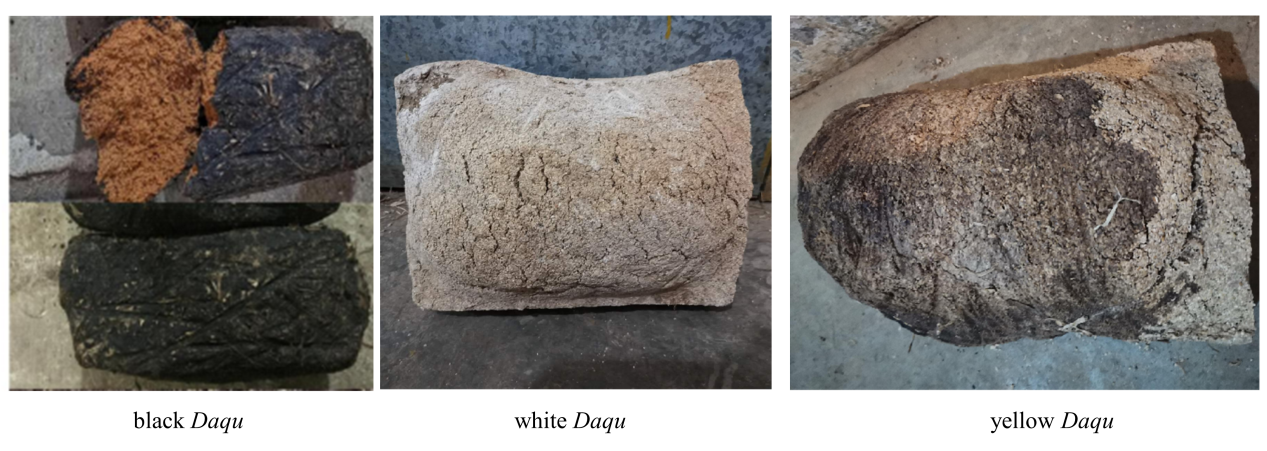


Fig. S1. The appearance of the three types of Moutai *Daqu*

*
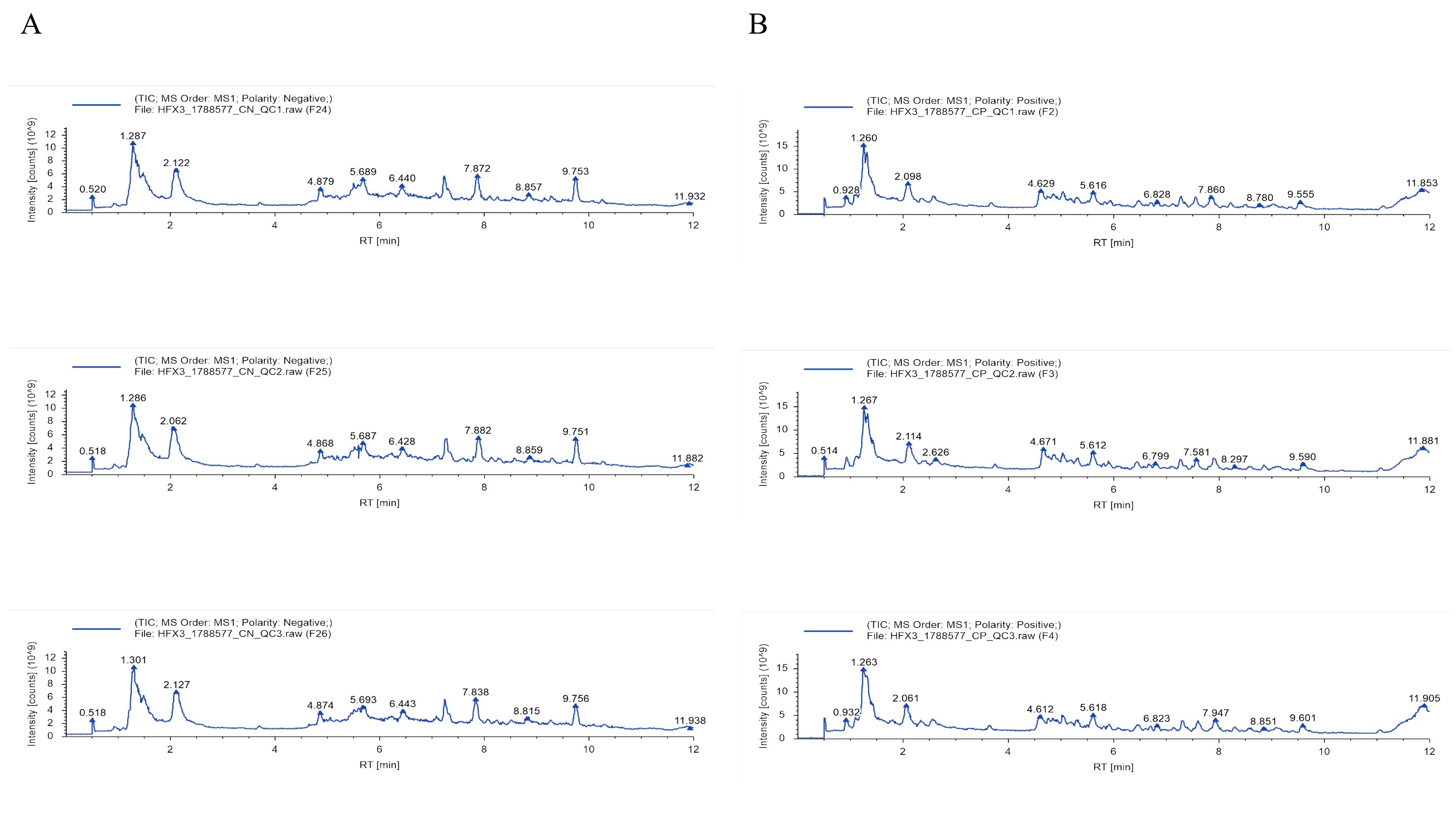
*

Fig. S2. Total ion current diagrams of QC samples in negative (A) and positive (B) ion modes





Fig. S3. Relative total content of amino acids, peptides, and analogues in the three types of Moutai *Daqu*





Fig. S4. Relative total content of fatty acids in the three types of *Daqu*

Table S1. Differences in enzymatic properties among the three types of *Daqu*

| Sample | Yellow *Daqu* | White *Daqu* | Black *Daqu* |
| --- | --- | --- | --- |
| Liquefaction power (U) | 0.22 ± 0.03^c^ | 0.46 ± 0.04^a^ | 0.29 ± 0.02^b^ |
| Saccharification power (U) | 127.20 ± 7.77^c^ | 207.15 ± 15.65^a^ | 157.98 ± 3.49^b^ |
| Fermentation power (g/0.5 g⋅72 h) | 0.15 ± 0.02^b^ | 0.14 ± 0.02^b^ | 0.19 ± 0.01^a^ |
| Esterification power (mg/50 g⋅7d) | 74.83 ± 5.18^b^ | 83.35 ± 2.45^a^ | 84.47 ± 2.45^a^ |

a, b, c is a significant marker and a indicates the maximum (Duncan-test *p* ＜ 0.01). The same mark letter indicates no significant difference, and different letter marks indicate significant difference.

Table S2. A total of 253 metabolites were detected by three types of *Daqu*

| name | Molecular Weight | retention times | relative quantitative values | | | | | | | | |
| --- | --- | --- | --- | --- | --- | --- | --- | --- | --- | --- | --- |
|  |  |  | yellow *Daqu* 1 | yellow *Daqu* 2 | yellow *Daqu* 3 | black *Daqu* 1 | black *Daqu* 2 | black *Daqu* 3 | white *Daqu* 1 | white *Daqu* 2 | white *Daqu* 3 |
| 8-iso Prostaglandin A2 | 316.20356 | 7.825 | 173039403.7 | 213133158.6 | 120216951 | 27327117.88 | 45178983.11 | 47157003.06 | 18084881.2 | 7960013.35 | 10759727.6 |
| Lathyrol | 334.21604 | 7.597 | 1366903.311 | 1734287.261 | 3667548.746 | 4787686.599 | 6438998.993 | 4435139.106 | 36999140.61 | 31223448.4 | 32185617.3 |
| N-[(-)-Jasmonoyl]-(L)-Isoleucine | 321.19517 | 7.243 | 11455776.02 | 12468172.11 | 8696812.847 | 20094264.93 | 23755816.39 | 28210606.68 | 5604864.994 | 4298089.344 | 13132832.06 |
| Pipecolic acid | 129.07836 | 1.594 | 1337582785 | 1891875720 | 1053206394 | 633081167 | 2764313396 | 981529217.5 | 239421686.9 | 141571075.4 | 341850282 |
| Methionine sulfoxide | 148.01852 | 1.265 | 4188643.864 | 2131734.448 | 4159575.019 | 283852.839 | 11306750.61 | 239442.2211 | 2064199219 | 1786345754 | 1704715784 |
| Muramic acid | 251.09909 | 1.242 | 2167084168 | 2925506640 | 1646754177 | 2269965248 | 465742243.5 | 2025415646 | 635280679.7 | 541870624.2 | 325995470.9 |
| N-Acetylneuraminic acid | 309.10428 | 1.312 | 282988998.4 | 473013721.8 | 342687377.1 | 654213434.8 | 318808379.4 | 479200110.2 | 177359727.2 | 153615034.7 | 236498995.7 |
| N-Acetylsphingosine | 341.29132 | 9.337 | 9464019.65 | 1531687.147 | 9239135.982 | 43347987.71 | 47372341.17 | 46336703.46 | 137682618.4 | 88506479.95 | 120222743.2 |
| 1-Linoleoyl-Rac-Glycerol | 354.27867 | 9.099 | 12757290.64 | 20057190.69 | 35192143.01 | 20083354.98 | 14691111.05 | 44051719.38 | 3342581.622 | 3270612.87 | 12281109.22 |
| Ala-trp | 275.12584 | 5.049 | 24925790.92 | 26960218.71 | 21434967.13 | 9234776.296 | 10169194.23 | 21735378.47 | 39317582.91 | 53366492.41 | 39966659.96 |
| D-glutamine | 146.06848 | 2.544 | 43500618.29 | 19357598.01 | 44081787.76 | 82668463.83 | 96744363.78 | 220795067.7 | 5272386.562 | 8240918.803 | 77241853.42 |
| Kinsenoside | 264.08336 | 1.568 | 17830995.56 | 20755196.73 | 15862080.68 | 5594532.288 | 15552969.95 | 9338236.716 | 13279397.26 | 11044460.76 | 8594849.716 |
| Corchorifatty acid F | 328.22727 | 6.317 | 371237790.9 | 302008145 | 232373622.6 | 814550172.9 | 754430221 | 653801840.6 | 346373651.5 | 217798943.4 | 232880383.3 |
| 9-HpOTrE | 310.21592 | 9.783 | 153267515.9 | 143063275.7 | 226158722.9 | 79831201.48 | 78320289.59 | 82668789.15 | 140044656.2 | 229268155.3 | 225893211.5 |
| Prostaglandin J2 | 316.20438 | 7.597 | 61521705.06 | 15123622.52 | 19112470.47 | 168056981.4 | 84144804.97 | 470902310.5 | 123188987.7 | 19439302.44 | 23505414.23 |
| trans-Petroselinic Acid | 282.25742 | 9.366 | 305169543.3 | 618424875.9 | 452318070.3 | 211285861.5 | 160911005.9 | 202657121.2 | 224738746.5 | 263465421.6 | 380361900.2 |
| N-Acetyl-D-alloisoleucine | 173.10636 | 5.575 | 958973317.9 | 926156764.2 | 424732065.3 | 1960044641 | 1930093118 | 2881141075 | 1230237982 | 340231267.1 | 931460880.3 |
| D-Saccharic acid | 210.0386 | 1.325 | 132482320.1 | 190318924.3 | 187069856.7 | 298358039 | 375662287.8 | 401898096.8 | 158609523.9 | 116301143 | 158720961.4 |
| N-Acetylalanine | 131.05899 | 2.527 | 993598145.2 | 492718125.9 | 456041730.4 | 1300740505 | 1214487952 | 2300724625 | 980794169.4 | 364856625.4 | 770159555 |
| Corticosterone | 382.18653 | 5.144 | 10449790.14 | 4833683.944 | 2860927.944 | 277489254.7 | 268060076.8 | 271544634.1 | 340973521.9 | 368151963 | 481641001.2 |
| LPE 15:0 | 439.27268 | 8.745 | 10347271.79 | 11556753.64 | 5950894.839 | 89995502.44 | 141146883.2 | 131785067.3 | 3105393.947 | 2391685.421 | 13657039.64 |
| (+/-)12(13)-DiHOME | 314.24739 | 7.285 | 10522247757 | 8367963898 | 8422201464 | 31691622820 | 38646950897 | 32463591676 | 10589436489 | 4667601119 | 5645673669 |
| Azelaic acid | 188.10602 | 5.877 | 769776681.5 | 501826976.4 | 517047816.8 | 1220476314 | 1687657085 | 1465359791 | 597822168 | 344982044.5 | 382294176.3 |
| 1-Stearoylglycerol | 358.30658 | 10.301 | 8052811872 | 5358533826 | 13070540390 | 3551568820 | 3755002176 | 1783876230 | 5168988724 | 6371927604 | 8220403307 |
| Sorbic acid | 112.05192 | 5.121 | 134292689.2 | 67206079.2 | 51684823.15 | 405268546.5 | 477429524.6 | 261207342.2 | 189688392.6 | 57485712.93 | 94070059.19 |
| N-Acetylornithine | 174.0996 | 1.398 | 485131626.6 | 270130714.6 | 315744248.4 | 951333232.5 | 566597889.5 | 710869423.8 | 622701496.6 | 212936450.9 | 426086658.6 |
| N-Acetyl-L-leucine | 173.1044 | 5.638 | 427061903.1 | 286936546.1 | 161894787.3 | 953331707.6 | 599209652.5 | 1376536926 | 550535133.2 | 139278775.6 | 287721335.2 |
| Betaine | 117.07829 | 1.273 | 20509692005 | 11859353197 | 16292501380 | 43122826746 | 62570557203 | 57848611900 | 38131689468 | 27266340991 | 35099674865 |
| Monoolein | 356.29086 | 9.762 | 1676603095 | 2320439476 | 4970115512 | 777148407 | 667992894.8 | 307536590.2 | 1442710447 | 2613132844 | 3459992166 |
| PC (14:0e/2:0) | 495.33002 | 8.968 | 18455732.1 | 19740791.97 | 8067767.571 | 55023987.68 | 40506620.92 | 53867047.76 | 25756834.71 | 9008225.69 | 12075780.62 |
| Oleoyl ethylamide | 309.30167 | 10.323 | 7073820855 | 3140169875 | 14508704549 | 14764479.26 | 18556111 | 23652919.4 | 4817962896 | 6273969657 | 6128770360 |
| MAG (18:3) | 352.25965 | 8.183 | 354642455 | 519449972.9 | 617375453.7 | 209410642.5 | 204014088 | 324951645.7 | 368611315.5 | 407444567.8 | 649511786.6 |
| Diosgenin | 396.29781 | 5.781 | 302555.1569 | 273015.8831 | 484390.9354 | 21121068.64 | 44573772.14 | 3811930.636 | 214686.1533 | 48567.96069 | 395727.0205 |
| Lithocholic Acid | 393.32253 | 9.796 | 2894652197 | 1117465011 | 1720023077 | 4207212350 | 4189426174 | 8600495286 | 2409184893 | 629505187.6 | 2082860794 |
| Coenzyme Q2 | 280.23012 | 9.76 | 104691774.7 | 97505492.6 | 124196899.9 | 58562252.38 | 69878486.55 | 76256029.14 | 90398258.15 | 104631072.7 | 100430796.7 |
| Linoleic acid | 280.23897 | 9.758 | 338947143.7 | 145443910.3 | 212011819.6 | 26629987.94 | 42394453.63 | 68747283.5 | 232633655.6 | 241688856.3 | 297506847.2 |
| N-Oleoyl Glycine | 339.27597 | 7.966 | 13638771.89 | 12693103.48 | 16259780.29 | 26294999.26 | 29587835.5 | 26056021.41 | 18782715.67 | 16087239.67 | 14323062.3 |
| 2-Hydroxyphenylalanine | 181.07309 | 5.619 | 54173370.02 | 85884233.59 | 64236100.5 | 813129078.2 | 17848058894 | 8306068298 | 150043765.9 | 159071470.6 | 131245598.2 |
| Lysope 14:0 | 425.25498 | 9.778 | 970478.8887 | 293924.3919 | 210437.2179 | 100938493.1 | 258072671.8 | 470994089.9 | 53837095.74 | 15289084.25 | 55226782.83 |
| L-Alanyl-L-Lysine | 217.14169 | 0.995 | 11123677.86 | 18304927.33 | 30001493.95 | 4947264.496 | 4707765.202 | 7728864.348 | 8047560.916 | 15952391.8 | 9266393.3 |
| DL-Panthenol | 205.13132 | 4.98 | 33256481.06 | 44804275.6 | 43103349.6 | 6131351.535 | 3143832.738 | 7930252.181 | 39578465 | 59376950.5 | 74048287.52 |
| 13(S)-HOTrE | 294.21814 | 9.768 | 382156264.1 | 258546547.6 | 239651395.3 | 126759258.5 | 112565751.9 | 109283252.3 | 152283719.8 | 131755402.5 | 158625918.8 |
| 6-Biopterin | 237.08665 | 1.209 | 280522397.5 | 221681214 | 252098901 | 190744655.5 | 269179835.3 | 467694158.4 | 202768880.3 | 144960197.5 | 236655773.7 |
| Guanine | 151.04876 | 2.988 | 108863470.5 | 86688681.66 | 91398603.99 | 209073031 | 563558025.3 | 200657499 | 206500916.5 | 129441541.9 | 275881793.7 |
| Sedanolide | 194.12974 | 6.482 | 197727788.4 | 144835684.6 | 140258192.5 | 49147713.9 | 79965401.03 | 215114671.8 | 34609898.82 | 24677060.87 | 30054717.74 |
| DL-Carnitine | 161.10438 | 1.15 | 781166709.1 | 934045675.7 | 980554356.2 | 1252198861 | 1048826385 | 1473749450 | 667393110 | 499981294 | 846238698.2 |
| N-p-Coumaroyl putrescine | 234.13595 | 4.885 | 2298486.58 | 1286679.553 | 1861823.491 | 4667615.838 | 2370212.549 | 5844555.796 | 43754022.39 | 28898673.7 | 33240016.83 |
| Tropine | 141.11462 | 9.156 | 44332412.32 | 31285949.02 | 31421690.2 | 15151575.77 | 16450866.73 | 19373360.47 | 61783859.98 | 64049518.79 | 49177418.55 |
| Senkyunolide A | 192.11419 | 7.474 | 24202337.99 | 23478956.7 | 22101093.68 | 29556577.65 | 39742126.71 | 41719548.09 | 17256554.06 | 11977564.91 | 23701960.38 |
| Levodopa | 197.06799 | 5.158 | 103141980 | 142987208.6 | 128232988.9 | 71146303 | 78578749.22 | 125205854.3 | 29423301.47 | 21385968.7 | 26347049.33 |
| N1-(2-amino-2-oxoethyl)-2-(isopropylthio)acetamide | 212.05756 | 5.427 | 405056061.7 | 270877066.9 | 348871319.4 | 253793600.3 | 347441272.5 | 299727189.5 | 62594340.32 | 25032492.29 | 50493989.14 |
| Kinetin | 237.06266 | 2.015 | 131813823.6 | 111330196.8 | 78068320.86 | 172157671.1 | 22218954.34 | 156100235.7 | 703934089.8 | 326685291.1 | 449106971.1 |
| Gluconic acid | 196.05925 | 1.306 | 6828277665 | 7966594203 | 9467501053 | 7929449131 | 5320160947 | 10124247511 | 5872732331 | 6205842643 | 5806949621 |
| (±)9(10)-DiHOME | 314.24724 | 7.487 | 5285556196 | 2550647646 | 2704579391 | 14630230.51 | 38695551.51 | 16490814.26 | 3323167403 | 1614875589 | 2550405680 |
| DL-Malic acid | 134.02229 | 1.529 | 6699225120 | 7890837295 | 4360008199 | 5561844988 | 2745482799 | 12038607548 | 7707163406 | 6452982144 | 6461081069 |
| Dulcitol | 182.08018 | 1.277 | 14252109330 | 35281143605 | 22110885223 | 13257809885 | 2425149148 | 12821621451 | 15259780541 | 23844041836 | 18866954474 |
| Sucrose | 342.11793 | 1.599 | 313504576.5 | 331750777 | 527320364.5 | 684932842.2 | 2111126502 | 1228776024 | 898049149.8 | 1323713497 | 577569451.2 |
| 4-Oxoproline | 129.04342 | 2.088 | 1.06E+11 | 75732106560 | 45587403528 | 40131505799 | 38919469053 | 75465738254 | 1.01E+11 | 59841142473 | 1.09E+11 |
| Elaidic acid | 282.25774 | 10.284 | 20426001123 | 9904079481 | 30775041570 | 7171065739 | 11300676333 | 4369826922 | 11656363011 | 15421161439 | 11541524792 |
| LPC 18:1 | 567.35858 | 9.323 | 58310188.5 | 82945728.61 | 25278968.8 | 162543213.6 | 125591368.5 | 132056058.6 | 134515132.1 | 24872132.88 | 30073007.05 |
| LPC 18:2 | 565.34175 | 8.683 | 125583543.5 | 126136192.9 | 20834928.47 | 344679197.2 | 354028109.6 | 242713101.9 | 253219632.3 | 43300598.61 | 50977067.22 |
| Galactinol | 342.11811 | 1.225 | 220582152.2 | 74383373.21 | 90827917.3 | 53470314.89 | 94275747.37 | 106298483.1 | 23482138.94 | 32339321.44 | 56653266.31 |
| 16-Hydroxyhexadecanoic acid | 272.23688 | 9.522 | 2887651870 | 1048598415 | 2525502837 | 2556669783 | 4783282064 | 5060941822 | 1693290285 | 951336284.7 | 1208992331 |
| Stachyose | 666.22644 | 1.393 | 18794627.19 | 12995296.52 | 14655554.24 | 19834358.04 | 17807352.4 | 35933462.94 | 18579882.81 | 9910154.665 | 13124789.55 |
| Fumaric acid | 116.01177 | 2.337 | 41594417.55 | 36224800.21 | 27140140.62 | 35328163.05 | 29581999.57 | 60884703.24 | 51394427.24 | 52829509.62 | 50352114 |
| LPC 16:0 | 541.34177 | 8.986 | 18751970.53 | 19963851.53 | 6672460.942 | 45169381.47 | 36741612.05 | 32037538.75 | 22783396.76 | 11654141 | 12341075.12 |
| Inositol | 180.0644 | 1.324 | 1352737998 | 1799953806 | 1199858069 | 1481674101 | 795743412 | 1061722780 | 1279522831 | 924561884.7 | 1153136377 |
| 5-OxoETE | 318.21932 | 7.906 | 85760589.13 | 40698111.78 | 197699728 | 914392917.8 | 874844916 | 730971240.5 | 66659212.27 | 2945488.942 | 1224641.358 |
| 6-Chloropurine | 154.004 | 1.544 | 212582739.8 | 291230215.9 | 193125757.3 | 183247401.2 | 236856075.8 | 200358647.6 | 184485476.7 | 256189794.4 | 173243774.4 |
| MGDG (2:0/18:2) | 604.35041 | 8.667 | 48340431.18 | 53023516.33 | 38698357.83 | 25516134.38 | 46213207.75 | 35638273.73 | 27373739.44 | 36003635.98 | 13960951.91 |
| (±)9-HpODE | 312.23195 | 9.779 | 1518371375 | 975580458.1 | 1310662291 | 386682295.3 | 318280291.9 | 355064007 | 2968071537 | 1488354314 | 2081313314 |
| δ-Gluconic acid δ-lactone | 178.0488 | 1.384 | 65900278.64 | 63502808.7 | 64666403.52 | 39386436.63 | 653938727.2 | 544867697.7 | 267120860 | 416856676.9 | 544709551.2 |
| 6-(Dimethylamino)purine | 163.08545 | 1.271 | 174956329.8 | 128287029.1 | 149717306.9 | 158140648.8 | 285469222.2 | 150713020.4 | 139478188 | 115043525.5 | 127669399.1 |
| L-Threonic acid | 136.03799 | 1.336 | 8370093037 | 6524304665 | 4542204485 | 9081158158 | 9213050769 | 10130408689 | 8041724193 | 4963048625 | 6812562016 |
| 10-Gingerol | 350.24558 | 10.285 | 816056348.5 | 285324455.6 | 908394932.4 | 370616840.1 | 578224830.6 | 258843166.5 | 700821993.5 | 924815581.2 | 759706127.3 |
| L-Cysteine-glutathione gisulfide | 426.08657 | 5.688 | 62274392.56 | 59537830.03 | 37950619.72 | 58176704.12 | 102385785.9 | 88218944.27 | 46377388.64 | 38761447.73 | 43964661.87 |
| FAHFA (18:2/20:4) | 582.46629 | 9.773 | 237015844.2 | 224216949.8 | 368795903.6 | 188720117 | 156778859 | 139766177.2 | 280843480.1 | 385822724.3 | 323769629.3 |
| Glutaconic acid | 130.02742 | 2.035 | 722414628.6 | 525838102.5 | 475927994.3 | 1081241588 | 2184922496 | 1051524812 | 849149811.3 | 248600630.7 | 314369798 |
| 3-Hydroxy-3-methylglutaric acid | 162.05359 | 2.936 | 710799032.4 | 504797642.6 | 300929809.4 | 434330427.2 | 1197084179 | 1019978751 | 599445789.6 | 228252498.7 | 376842938.4 |
| trans-zeatin 9-O-glucoside | 381.16637 | 5.009 | 14609266.08 | 6910528.189 | 7069054.314 | 12318138.71 | 24872748.38 | 19528829.85 | 8401205.432 | 496079.8025 | 4816214.229 |
| D-α-Hydroxyglutaric acid | 148.03809 | 1.862 | 1243497926 | 893525526.2 | 953266653.9 | 700904106.1 | 2161282530 | 1190687049 | 458077172.9 | 172950825.3 | 245616869.5 |
| β-D-Glucopyranuronic acid | 194.04357 | 1.329 | 421688960.5 | 420648726.9 | 345944617 | 418783098.5 | 521147754 | 555573403.6 | 381983391.8 | 221802397.5 | 411367280.7 |
| 3-Phenyllactic acid | 166.06391 | 5.713 | 5387217281 | 2175744509 | 2034488997 | 10838452019 | 10856814752 | 7464530365 | 8060492653 | 1559283691 | 1579286812 |
| Pentadecanoic Acid | 288.23179 | 6.535 | 335774094.7 | 170331080.9 | 922639012.5 | 196525012.7 | 436402578.9 | 113503097.2 | 353426353.4 | 114747455.4 | 127492208.8 |
| L-Serine | 105.04328 | 1.198 | 564037722.3 | 682476342.4 | 522662725.7 | 337127149.5 | 252047601.1 | 642211957.3 | 810585391.7 | 769388288.6 | 890813558.3 |
| (±)9(10)-EpOME | 342.24224 | 7.921 | 554252005.4 | 138449269.3 | 290307871 | 163119011.4 | 28203936.01 | 29337133.41 | 277959889 | 135452188.5 | 227185186.3 |
| Thioctic acid | 206.04347 | 1.397 | 87190640.04 | 58341724.36 | 164907772.6 | 59237001.47 | 60091795.23 | 38464308.42 | 81252518.8 | 51575532.1 | 69593580.61 |
| Malonic acid | 104.01159 | 1.712 | 234585015.8 | 150140371.2 | 379984764.2 | 247602442.7 | 120941767.7 | 314038126.6 | 100680073.2 | 79330351.63 | 143948028.8 |
| D-Mannitol 1-phosphate | 262.04668 | 1.488 | 578770261.2 | 1059944062 | 665833115.3 | 468071491.2 | 105820001.1 | 621557418.3 | 408454156 | 425225000.6 | 500246986.5 |
| FAHFA (18:1/20:3) | 586.49797 | 10.288 | 148345581 | 148427052.1 | 203588453.9 | 76530692.76 | 138655094.5 | 49797562.7 | 137300202.6 | 225479761.7 | 169187660.6 |
| Dehydrodiisoeugenol | 326.15131 | 7.82 | 68354005.61 | 31348066.21 | 32361874.08 | 41829064.37 | 51986436.45 | 70062819.52 | 79666771.04 | 33157157.46 | 35255327.93 |
| Glycerol 3-phosphate | 172.01456 | 1.498 | 332037604.6 | 400089973.4 | 315839191.8 | 265970867.9 | 283956644 | 374776999.9 | 383420998.2 | 309860906.6 | 312520939.9 |
| 8-O-acetyl shanzhiside methyl ester | 448.15797 | 9.556 | 7853881.669 | 20371125.96 | 33340836.64 | 4428178.273 | 5150732.347 | 11719770.14 | 13089770.29 | 10090585.64 | 18576368.96 |
| Schizandrol A | 432.21282 | 7.908 | 24243074.5 | 29241972.22 | 30631704.55 | 6999777.913 | 16286039.94 | 17015849.88 | 22389496.86 | 35099830.36 | 28047314.96 |
| 17(S)-HpDHA | 360.23217 | 9.291 | 28417615.2 | 29645709.78 | 13833312.99 | 16331305.73 | 37482903.64 | 72483421.88 | 13511037.01 | 1688308.724 | 11161427.07 |
| DL-4-Hydroxyphenyllactic acid | 182.05901 | 5.183 | 644835262.5 | 427458546.1 | 426241575.8 | 591620693.8 | 2282532024 | 96795963.79 | 744605334.6 | 323506955.9 | 351983837.7 |
| 3-Methyladipic acid | 160.0745 | 5.443 | 58061904.99 | 42133639.7 | 44110240.74 | 67945797.19 | 108646685.4 | 66465527.83 | 52149413.35 | 30819189.21 | 35426991.65 |
| 6-Benzyladenine | 225.10129 | 5.695 | 1621496257 | 698557220 | 586995795.7 | 41196994.87 | 39170963.34 | 31098695.47 | 1278936035 | 339558300.3 | 892101652.8 |
| Diosbulbin B | 344.12557 | 4.955 | 14582319.01 | 5553505.345 | 4627662.925 | 31043741.39 | 30478578.69 | 46329195.68 | 16634123.04 | 12299497.42 | 16357714.76 |
| N-Acetyl-α-D-glucosamine 1-phosphate | 301.0578 | 1.495 | 62420144.28 | 42727836.92 | 67100721.19 | 43720853.43 | 41908102.13 | 58265863.64 | 59914745.95 | 46491741.15 | 45162594.87 |
| 4-(hydroxymethyl)benzoic acid | 152.04823 | 5.54 | 1040130595 | 477193479.2 | 512493952.8 | 1098751741 | 1868032965 | 1077350022 | 707850521.9 | 285700448.8 | 608220361.9 |
| 7-(2-Hydroxyethyl)theophyline | 224.09081 | 1.253 | 50560583.37 | 68400321.31 | 45663473.1 | 42831949.45 | 48354494.53 | 46514309.82 | 41738593.34 | 74459761.04 | 34874234.15 |
| FAHFA (16:0/18:2) | 534.46614 | 10.098 | 28610269.55 | 22690247.59 | 43112054.27 | 20222395.97 | 14898788.38 | 10846386.51 | 21641413.01 | 26981809.12 | 39525981.27 |
| Citrulline | 175.09655 | 1.247 | 140589187.7 | 66281819.78 | 31861204.63 | 95211867.67 | 107161514.4 | 262580242.8 | 73142121.31 | 17148185.79 | 180822422.8 |
| 2'-Deoxyinosine | 252.08595 | 1.946 | 527945067.9 | 707903527.4 | 466741025 | 585961028.5 | 609094043.5 | 776235179 | 535740995 | 549443823 | 522393300.3 |
| 11(E)-Eicosenoic Acid | 310.28922 | 11.051 | 725125498.9 | 152355032.2 | 1046228876 | 76423138.75 | 151873344.4 | 19748604.84 | 237819477.8 | 224992786.9 | 306355123.2 |
| Przewaquinone A | 310.11992 | 4.927 | 6705369.753 | 70001567.94 | 2320521.377 | 28078969.71 | 22281455.96 | 8038142.513 | 34243972.64 | 3513076.136 | 4099191.56 |
| trans-10-Heptadecenoic Acid | 268.24171 | 9.903 | 123421834.5 | 73662108.76 | 163924922.3 | 69240990.52 | 117141803.2 | 56854495.35 | 62763634.91 | 76418210.17 | 76538919.32 |
| Cordycepin | 251.10166 | 1.253 | 222347778.1 | 335455200.3 | 168392411.9 | 235785606.1 | 81723762.15 | 203186999.5 | 166611732.7 | 200407042.1 | 258009988.9 |
| Taxifolin | 304.06016 | 5.504 | 4086022.067 | 4156671.674 | 3356519.066 | 633320.2896 | 1734422.794 | 627324.7988 | 3066118.327 | 3366098.006 | 2838517.708 |
| Xanthosine | 284.07735 | 4.604 | 42548720.83 | 45148279.35 | 15184230.55 | 19596629.29 | 18981944.2 | 30617603.95 | 31909514.14 | 8497450.423 | 22651824.87 |
| Neocryptotanshinone | 314.15051 | 7.308 | 15223035.09 | 3878093.872 | 5416313.315 | 16749846.19 | 14241557.83 | 21639459.94 | 4917913.197 | 686119.0779 | 5454610.609 |
| Meso-erythritol | 122.0585 | 1.311 | 3310960155 | 2502046607 | 1994570048 | 2309359409 | 1718021988 | 3657846296 | 3620896587 | 2802036295 | 2134497696 |
| 10-Formyl-THF | 473.16644 | 8.919 | 8641067.74 | 7444128.338 | 5717585.221 | 4149082.021 | 8202138.335 | 8112125.28 | 7364108.679 | 6357359.504 | 4906586.608 |
| N7-Methylguanosine | 299.12315 | 1.263 | 41808075.52 | 43819775.59 | 27449280.94 | 24687547.6 | 20718783.14 | 27378465.32 | 36830090.53 | 28588957.26 | 38048710.37 |
| Tigloylgomisin H | 500.24266 | 8.149 | 11612177.71 | 2505171.655 | 3862775.22 | 9663003.765 | 9540759.196 | 10607366.98 | 13186587.5 | 2208766.431 | 3035037.472 |
| Quercetin | 302.0413 | 5.64 | 12094898.28 | 6819800.585 | 7207266.365 | 7661540.146 | 6237302.665 | 8556683.539 | 8568437.641 | 12391268.01 | 13484984.49 |
| Orotidine | 288.05846 | 1.227 | 4342823.391 | 3995588.69 | 2919751.528 | 3900135.513 | 2704246.22 | 5189790.858 | 5037489.982 | 6458306.027 | 4855505.214 |
| 2-(Dimethylamino)Guanosine | 311.12358 | 1.24 | 30514824.11 | 44654793.22 | 25897033.82 | 26396205.94 | 11121697.59 | 36550574.47 | 21662558.9 | 24422641.76 | 31364120.03 |
| Danshenol C | 336.13655 | 5.204 | 145560496.8 | 82558689.92 | 120482844.9 | 51474711.83 | 80873245.04 | 111396891.4 | 143148456.2 | 83666616.89 | 137728490.7 |
| FAHFA (18:2/3:0) | 352.26332 | 10.656 | 409562119.5 | 405586774.7 | 326232618.2 | 798834720.5 | 377303718.4 | 901041363 | 625123189.1 | 353099392.8 | 354790770.1 |
| Isorhamnetin | 316.05902 | 2.321 | 192287764.4 | 150981709.5 | 75516576.79 | 62868451.21 | 47099305.55 | 119534033 | 224183116.6 | 170433815.9 | 175407875.5 |
| Vincamine | 354.19571 | 8.302 | 63183662.04 | 62225658.63 | 72103911.9 | 79658300.47 | 81390757.48 | 74739476.3 | 69017308.36 | 51421823.89 | 63180386.44 |
| Talatisamine | 421.28334 | 9.868 | 58328035.56 | 34688091.42 | 65928744.33 | 42728308.89 | 72964035.9 | 62852087.83 | 27813320.63 | 7814376.942 | 17713364.58 |
| Adenosine5-phosphosulfate | 427.02141 | 1.324 | 10034135.87 | 2024863.687 | 9441912.088 | 10922453.19 | 3199078.066 | 11312628.28 | 5921379.185 | 3614475.43 | 5646955.995 |
| Citric acid | 192.02825 | 2 | 3894686551 | 3862776230 | 1976680699 | 2006146764 | 2848862043 | 5865725606 | 2998237522 | 778267480.7 | 2061868431 |
| Allantoin | 158.04492 | 1.321 | 343916645 | 293972862.9 | 395519545.2 | 272585034.4 | 213545363.6 | 282705901 | 208857901.4 | 275709421.3 | 270737075.2 |
| 2-Isopropylmalic acid | 176.06959 | 5.462 | 2479199987 | 116577149.8 | 1073987805 | 5051920471 | 14901551284 | 7139733980 | 2561810434 | 316844803 | 841296260.2 |
| LPC 14:0 | 513.31034 | 8.373 | 2249870.786 | 1702765.247 | 1008938.795 | 2235327.161 | 6728677.628 | 2857672.025 | 2200887.948 | 2229869.149 | 1653296.346 |
| DL-Stachydrine | 143.09393 | 1.384 | 618133554.7 | 1419685024 | 1042734429 | 1569814438 | 11428058448 | 26030363882 | 289792848.8 | 1378706680 | 821599033.7 |
| L-Pyroglutamic acid | 129.04204 | 2.093 | 57021053292 | 44197827245 | 29092483938 | 27667100967 | 23205773761 | 44570605269 | 55022889141 | 30326011843 | 53238352592 |
| Indole | 117.0573 | 5.042 | 54695116.96 | 32398505.11 | 23164780.2 | 75883234.79 | 73102662.86 | 110800439.8 | 44516455.01 | 29523589.48 | 45277937.14 |
| Trigonelline | 137.04704 | 1.67 | 274575146 | 257597098.9 | 323254582.4 | 390070482.9 | 570560946.7 | 541807285.6 | 354501235.7 | 249832405.7 | 372475501.3 |
| Stearamide | 283.28629 | 10.168 | 4032924148 | 1824695338 | 8762229216 | 1540686429 | 1107264658 | 405278665.4 | 2952963340 | 4190870972 | 3988565964 |
| Uracil | 112.02678 | 2.081 | 1980207441 | 1734993461 | 1181276674 | 2892980727 | 3136232321 | 3178697384 | 1856966278 | 806497716.7 | 1481769430 |
| Cuminaldehyde | 148.08811 | 6.837 | 244932870.3 | 371021716.5 | 455197168.1 | 355496776.7 | 519556508.8 | 588537245 | 401814580.2 | 515205439.3 | 701263663.1 |
| Thymine | 126.04243 | 4.774 | 2441117052 | 1977446910 | 1588124973 | 2469227772 | 2294159010 | 2773689338 | 1916520062 | 1351338947 | 1411744225 |
| D-Serine | 105.04213 | 1.191 | 821318211.3 | 975164278.3 | 797540081.5 | 497160402.2 | 327266758.8 | 889250502.1 | 1043364462 | 1195763925 | 1326048877 |
| Acetophenone | 120.057 | 6.493 | 596898592.2 | 703330545.8 | 440296523.5 | 25102989.56 | 5198804.492 | 27040496.69 | 140539454 | 174748194.8 | 149357602 |
| Palmitoyl ethanolamide | 299.28095 | 9.315 | 207482468.1 | 128383633 | 244550553.1 | 369882259.7 | 314479202.5 | 327709064.4 | 179877924.3 | 69106202.21 | 142205304.5 |
| 4-Hydroxybenzaldehyde | 122.03622 | 2.054 | 1082171146 | 1002140229 | 712503262.5 | 484616725.2 | 656164242.3 | 808568039.1 | 942383437.7 | 740274908.9 | 832052525.7 |
| N8-Acetylspermidine | 187.1676 | 0.996 | 90664782.56 | 94643853.16 | 84887313.11 | 89387875.79 | 96492201.27 | 111059103.1 | 85324682.33 | 66262857.32 | 61265069.87 |
| Indole-3-lactic acid | 205.07308 | 5.455 | 63355756.49 | 45345975.91 | 32825129.79 | 196834623.7 | 224970184.6 | 133476487.2 | 107425152.6 | 38925881.81 | 36920200.77 |
| L-Kynurenine | 208.0838 | 3.708 | 549155057.7 | 217630935.2 | 239650286.1 | 230677756.4 | 32286625.84 | 158133867.2 | 297060543 | 111274552.2 | 36535777.42 |
| Acetylcholine | 145.10964 | 1.823 | 30302694.89 | 36136266.16 | 35198839.91 | 85896481.81 | 690681737.5 | 331014992.2 | 35325161.88 | 27808166.77 | 32511119.69 |
| 6-Methylquinoline | 143.07281 | 4.948 | 117060761.8 | 35983123.29 | 17930110.62 | 205931577.4 | 472347981.9 | 2467880366 | 794403763.3 | 569861493.7 | 611203284.4 |
| Imidazoleacetic acid | 126.0423 | 1.218 | 412648709.1 | 360877245.6 | 252469953.7 | 437336209.9 | 523948850.9 | 453079725.7 | 356906845.1 | 242550403.8 | 267460813.7 |
| 4-Acetamidobutanoic acid | 145.07318 | 3.156 | 232898417.6 | 262393460.5 | 268285126 | 474598455.8 | 324933313.4 | 449660319.1 | 231185595.2 | 95571466.44 | 182542779.1 |
| 8-Hydroxyquinoline | 145.05214 | 5.227 | 140612802.9 | 99025230.39 | 53096718.94 | 171170558.6 | 177041028.9 | 225206329 | 69326432.28 | 27602277.4 | 50163676.97 |
| Progesterone | 314.22305 | 9.695 | 92047068.53 | 16712509.26 | 4410453.31 | 13976737.44 | 77952452.53 | 11642262.85 | 40019025.25 | 3759504.712 | 24222816.7 |
| Citrinin | 250.08313 | 5.408 | 4083432.979 | 2005549.028 | 15316223.05 | 5825976.942 | 30152157.42 | 14529927.68 | 141763393.9 | 14477094.83 | 5763136.577 |
| 5-Hydroxyindole-3-acetic acid | 191.05736 | 5.387 | 47186597.31 | 47801290.38 | 34226796.2 | 37585624.66 | 12362482.37 | 25624387.32 | 257463914.8 | 55559349.43 | 80415256.16 |
| Serotonin | 176.09412 | 3.611 | 86154851.8 | 14484441.63 | 37949036.42 | 8872541.971 | 14124218.46 | 48678475.51 | 11970139.93 | 12705908.69 | 11893748.89 |
| DL-2-(acetylamino)-3-phenylpropanoic acid | 207.08857 | 5.679 | 692432569.8 | 292450415.1 | 228323632.2 | 1040493969 | 631826815.8 | 1311316952 | 554172833.2 | 109203161.1 | 382116879.1 |
| DL-Tryptophan | 204.08887 | 5.041 | 298472330.5 | 250468967.5 | 300511163.4 | 2263592333 | 2304222788 | 3509211235 | 671116422.4 | 115672766.5 | 654083473.6 |
| Asparagine | 132.05283 | 1.196 | 434668913.8 | 629576346.6 | 414483345.9 | 227401132.4 | 204319011.3 | 592882554.3 | 630137835.9 | 886357578.3 | 824891343.9 |
| Hypoxanthine | 136.03792 | 1.859 | 881883597.8 | 811861131 | 569064737.7 | 1437730382 | 1969606527 | 1767709857 | 778695943.9 | 374738545.7 | 630151474 |
| Adenosine | 267.09552 | 2.444 | 698729732.9 | 2201653529 | 1036808778 | 130928393.1 | 365730734.5 | 594582306.4 | 831780555.4 | 881449389.4 | 829980609.8 |
| L-Glutamic acid | 147.05247 | 1.232 | 8614157601 | 7500443157 | 6285104808 | 7570373521 | 7032773240 | 8312116405 | 8182666654 | 6196132067 | 6519951819 |
| DL-Arginine | 174.11086 | 1.068 | 1352550946 | 2595966871 | 1549722901 | 1098614489 | 828045277.3 | 1241847737 | 2904574941 | 2569079600 | 1774169942 |
| L-Phenylalanine | 165.07831 | 4.626 | 26708239924 | 15795507883 | 12300170813 | 13308127549 | 13551172042 | 27951327595 | 20786246487 | 15807001818 | 22905522937 |
| Oleoyl ethanolamide | 325.29664 | 9.518 | 363297299.7 | 275048207.4 | 451559454.9 | 698943519.9 | 688179548.8 | 472705170.2 | 316214184 | 140056051.6 | 315192444.3 |
| Xanthine | 152.03274 | 2.05 | 743609918.1 | 565936367.3 | 419620899.1 | 1428152630 | 2320510323 | 1861204287 | 523157122.2 | 215470014.2 | 416101679.1 |
| Nicotinic acid | 123.03156 | 1.784 | 2263140003 | 1934352628 | 2196719854 | 2208093773 | 3547153291 | 3150552028 | 1869928423 | 967461268.8 | 1328114689 |
| Pantothenic acid | 219.10973 | 4.983 | 215543359.4 | 119886805.5 | 169896322.9 | 285116605.2 | 387154073.4 | 346500872.8 | 146919432.6 | 100251703.4 | 97922108.91 |
| Uric acid | 168.02759 | 1.745 | 1194141360 | 1083628521 | 1202359493 | 1410845103 | 1326850454 | 413968054.1 | 1363484317 | 851989722.9 | 699202161.7 |
| L-Histidine | 155.0688 | 1.053 | 399294717.1 | 407029647.3 | 265499483.3 | 323491402.1 | 238843541 | 439469215.8 | 498283379.6 | 315205937 | 253907651.6 |
| Adenine | 135.05396 | 1.414 | 523697543.3 | 469174513.5 | 267180025.8 | 621506971.2 | 780292997 | 860189210.3 | 800519812.3 | 257648569.6 | 456762540.1 |
| Thymidine | 242.08918 | 4.775 | 106051314.6 | 97340161.58 | 67332951.69 | 48936769.6 | 108856409.7 | 62336787.93 | 62460396.35 | 25587693.1 | 45329603.06 |
| N-Acetyl-DL-glutamic acid | 189.0628 | 2.194 | 1001211280 | 337206070.7 | 285101921.4 | 907662093.1 | 514212588.8 | 567722403.9 | 291316404.1 | 439590244.2 | 176551465 |
| Kynurenic acid | 189.04181 | 5.661 | 462006796.3 | 231520224.5 | 424404754.2 | 475127756 | 1092851255 | 468729104.6 | 273994163.9 | 147376114.4 | 197003060 |
| Oleamide | 281.27033 | 9.603 | 57093452970 | 46931249271 | 1.1E+11 | 56749750250 | 33411773310 | 28724577245 | 54839299201 | 68655894290 | 69808339563 |
| Choline | 103.09914 | 1.119 | 11401414021 | 10452919923 | 9298534620 | 11636813711 | 11117673928 | 17483599042 | 12785980339 | 7142956157 | 12680555921 |
| Linoleoyl ethanolamide | 323.28074 | 9.015 | 1748875624 | 1160486260 | 1682148654 | 3401901544 | 3250837095 | 3629391718 | 1634434914 | 873506666.7 | 1661417468 |
| Hexadecanamide | 255.25493 | 9.416 | 11460424416 | 9011503081 | 23862614940 | 12014529872 | 7255474582 | 6925657494 | 11761127678 | 13872237450 | 13874267715 |
| 1-Palmitoylglycerol | 330.27528 | 9.57 | 6823423488 | 5629945215 | 8673585488 | 5124584730 | 5328811887 | 3335646394 | 4845896121 | 5837038262 | 8346896234 |
| 3-methyl-5-oxo-5-(4-toluidino)pentanoic acid | 257.10159 | 1.244 | 2218676865 | 7460029309 | 5501199516 | 816478610.7 | 1097121377 | 1184284884 | 4226029208 | 4824376895 | 2910528320 |
| PC (16:2e/2:0) | 519.32973 | 8.678 | 137599580.5 | 123942257.6 | 16644093.53 | 355043112.8 | 312090222.1 | 332994911.9 | 361788290.8 | 48163949.09 | 58080304.25 |
| Prolylleucine | 228.14629 | 1.431 | 65665630.39 | 35074238.69 | 121674037.9 | 140740917.1 | 200265256.1 | 156730971.5 | 90251949.9 | 88968463.72 | 65406513.61 |
| L-Iditol | 182.07809 | 1.282 | 2971782764 | 8414765355 | 4426046978 | 2368307771 | 441736327.5 | 1796129349 | 2767477771 | 4532320745 | 3193968274 |
| MGMG (18:2) | 516.32729 | 8.883 | 1663950.215 | 19788801.35 | 15527650.98 | 1325392.976 | 2952305.478 | 12810566.46 | 3417041.289 | 13916191.57 | 12498267.53 |
| 4-Guanidinobutyric acid | 145.08439 | 1.585 | 533558793 | 648172212.2 | 667918981.5 | 481611489.7 | 313693890.1 | 740753963.8 | 838500328.6 | 720154136.6 | 677165874.1 |
| N6-Me-dA | 265.11477 | 1.185 | 772437546.9 | 1463928604 | 928495526.3 | 354720331.1 | 134091071.9 | 959640625.3 | 475250145.8 | 837709019.9 | 887285136.5 |
| Phosphocholine | 183.06514 | 1.228 | 361565384.2 | 358077418 | 188102735.1 | 200491964 | 263507565.5 | 83196710.55 | 440627744.4 | 238096035.1 | 149492721.3 |
| N-Caffeoyl putrescine | 250.1307 | 4.834 | 58363111.58 | 28707625.88 | 7829516.088 | 17075392.07 | 30171814.05 | 88035007.29 | 17211974.37 | 5507482.753 | 37137303.92 |
| N-(4-butyl-2-methylphenyl)-N'-[4-(4-methylpiperazino)phenyl]urea | 380.25212 | 6.369 | 49736888.85 | 42214405.95 | 31044352.45 | 14504724.31 | 15104828.99 | 23827681.41 | 37784201.89 | 27697842.81 | 54074979.46 |
| Sinapinic acid | 206.05694 | 5.657 | 6023142.871 | 5851661.435 | 4723647.068 | 27813729.26 | 37886944.86 | 30699115.9 | 7783364.718 | 17705682.24 | 14517365.87 |
| Ambroxane | 236.21286 | 8.166 | 341372169.3 | 321655368.7 | 394509779.8 | 261692633.2 | 275223525.2 | 374723157.8 | 312953482.6 | 247518109 | 315257472.6 |
| α-Linolenoyl ethanolamide | 321.26516 | 7.402 | 26852767.42 | 16587669.61 | 21908643.89 | 75769879.64 | 115282655.2 | 102089175.5 | 107789371.5 | 36325151.1 | 89973672.93 |
| Dihydrothymine | 128.05791 | 1.245 | 44811276.55 | 49446297.44 | 45100060.02 | 88219887.21 | 59948608.2 | 93091910.57 | 55058924.34 | 40608613.66 | 43054800.57 |
| Dihydrojasmone | 166.13498 | 6.463 | 121943697.6 | 87138016.89 | 84221439.42 | 153653158.6 | 176940257.1 | 135336345.9 | 94441279.77 | 59464618.55 | 64140117.09 |
| Histamine | 111.07915 | 0.951 | 184029243.3 | 261198657.9 | 123905954.3 | 170660872.6 | 115406200.3 | 127495001.2 | 156552344 | 64247282.54 | 80011721.96 |
| 4-oxododecanedioic acid | 266.11399 | 6.49 | 5555131.375 | 4296863.458 | 3822278.974 | 28583575.25 | 32492263.62 | 23633817.47 | 106155910.6 | 109716554.1 | 108086787.3 |
| Diaminopimelic acid | 190.0944 | 1.311 | 224849301.5 | 170413296.9 | 138186624.3 | 188272246.7 | 137955420.5 | 223112997.2 | 458146099.2 | 184803115.5 | 295927174.1 |
| Olivetol | 180.11419 | 6.2 | 30508024.34 | 29638861.23 | 39099835.21 | 39784170.96 | 47327717.39 | 86550422.45 | 28743951.54 | 22535203.61 | 47876558.09 |
| Glycyl-L-leucine | 188.11519 | 2.611 | 58254089.3 | 53977741.82 | 33897821.25 | 29105133.82 | 40564679.94 | 71945387.19 | 62841592.05 | 59845619 | 61867534.76 |
| Muscone | 238.22837 | 9.57 | 128920687.6 | 108015795 | 136083056 | 96493620.2 | 108040298.8 | 68461892.67 | 80102345.17 | 100718703.4 | 139501445.8 |
| N6-Acetyl-L-lysine | 188.11509 | 1.434 | 1240751380 | 551394214.2 | 2124876033 | 1824004349 | 4943954650 | 1230307701 | 856947405.6 | 197335212.4 | 771205825 |
| 3-Methylcrotonylglycine | 157.07324 | 4.887 | 732756252.2 | 394179471.1 | 204184220.8 | 683981658.5 | 914145704.5 | 1037357544 | 572691138 | 213888977.1 | 358910550 |
| 1-Phenyl-3-methyl-5-pyrazolone | 174.07851 | 5.043 | 81061186.78 | 33320811.57 | 10347900.42 | 63419443.54 | 162232922.7 | 112425343.5 | 41524836.15 | 10302991.38 | 27125849.7 |
| 3-Methoxy prostaglandin F1α | 368.2547 | 9.283 | 100239941.6 | 89182792.95 | 62778969.16 | 97693406.01 | 66155813.83 | 150495583 | 85825957.53 | 46903089.63 | 81948910.76 |
| L-Saccharopine | 276.13069 | 1.204 | 197930357 | 189261953.9 | 147358695.4 | 183158583.3 | 226659870.3 | 225710272.2 | 184322965.8 | 95746148.11 | 176157031.3 |
| TriacetonaMine | 155.13025 | 9.148 | 48122094.81 | 63279499.75 | 141423823.7 | 52129607.96 | 55346945.83 | 56924940.02 | 79485910.62 | 49161721.35 | 54507558.71 |
| C-pentosyl-apeignin O-feruloylhexoside | 740.19175 | 5.391 | 4075707.64 | 3529786.083 | 3915613.839 | 5988585.201 | 7264515.765 | 7301075.964 | 3520321.505 | 3213906.449 | 3140186.683 |
| 3-[4-methyl-1-(2-methylpropanoyl)-3-oxocyclohexyl]butanoic acid | 268.16616 | 6.274 | 149436487.7 | 156771622.4 | 124676140.8 | 4287318.018 | 14209081.81 | 12381288.27 | 53606211.06 | 29439554.86 | 103013968.4 |
| Methyl palmitate | 270.25432 | 9.57 | 71664180.45 | 60407731.7 | 80200884.88 | 50654736.5 | 58437944.4 | 38593319.43 | 46147010.2 | 56325636.28 | 84143015.39 |
| Tetramethylpyrazine | 136.09944 | 5.628 | 48983540.65 | 18214811.14 | 25260282.33 | 408119010.8 | 941762350.2 | 679690044.5 | 46736153.27 | 11443807.87 | 43907211.27 |
| RMH | 464.19864 | 1.416 | 10386238.88 | 10809152.7 | 8437027.22 | 9380885.483 | 9573244.931 | 2110834.709 | 13261612.61 | 5225529.858 | 7680406.319 |
| Cytosine | 111.04269 | 1.162 | 199726100.1 | 226139659.2 | 149324592.8 | 116406668.4 | 163323392.4 | 292611796.6 | 197369106.4 | 159286795.7 | 249288579.5 |
| Pyridoxamine | 168.08914 | 4.802 | 910309364.5 | 57576071.54 | 49994236.34 | 641879281.8 | 909458649.2 | 421427618.7 | 710776465.1 | 381011505 | 572105155 |
| octadec-9-ynoic acid | 297.26538 | 6.288 | 9041603.33 | 6489189.185 | 10295908.68 | 3335381227 | 2228388920 | 2368421499 | 21437969.92 | 30317998.78 | 37312894.19 |
| N6-Succinyl Adenosine | 383.10598 | 4.989 | 114240962.6 | 103370596.5 | 89786367.42 | 49357223.64 | 82330211.33 | 96504412.86 | 78260619.3 | 56066649.01 | 41256511.91 |
| N-Phenylacetylglycine | 193.07308 | 5.328 | 83510175.65 | 93468800.97 | 59205892.58 | 41652282.07 | 40228929.9 | 80938537.07 | 205662516 | 49292638.25 | 87623132.48 |
| WLH | 454.23091 | 8.166 | 41769951.65 | 2368681.387 | 6723475.387 | 17454514.46 | 24691472.62 | 28099713.13 | 28232041.77 | 3220344.63 | 3259739.79 |
| Gamma-Glu-Leu | 260.13598 | 5.263 | 1271362.385 | 77959881.57 | 71454084.58 | 136664600.7 | 438740122.7 | 242149160.8 | 99721651.48 | 34381237.53 | 82565599.23 |
| Pyridoxal | 167.05489 | 3.163 | 37964798.94 | 33586717.57 | 28032753.72 | 137127121.4 | 112405798.6 | 300822473 | 16735461.61 | 16983737.49 | 26119671.31 |
| Nobiletin | 402.13 | 5.668 | 15226161.97 | 8626673.7 | 14158118.94 | 11412134.72 | 15884922.61 | 15124679.3 | 17115741.71 | 10924436.75 | 13326940.53 |
| N2-Methylguanosine | 297.10593 | 4.735 | 15946744.9 | 16514956.29 | 20592359.09 | 1329624.069 | 1260924.537 | 1619375.872 | 13773552.55 | 11832447.54 | 18424010.94 |
| Peimisine | 427.30669 | 10.044 | 874009096.3 | 306094990 | 608055550.9 | 1008248922 | 2348195054 | 2703918122 | 464127940.5 | 111734278.1 | 632207369 |
| N-Acetyltryptamine | 202.10962 | 5.734 | 50563535.16 | 11191902.63 | 5506995.652 | 64544918.55 | 67256811.17 | 539774888.5 | 34119717.33 | 16760412.1 | 39908442.12 |
| Octanedioic acid | 174.08865 | 5.435 | 21502534.47 | 12883548.26 | 14091100.25 | 41952927.69 | 29873859.63 | 29121885.93 | 20599532.36 | 7253023.562 | 9994345.846 |
| 7-alpha-carboxy-17-alpha-carboxyethylandrostan lactone phenyl ester | 432.28517 | 8.702 | 46234958.45 | 9247169.675 | 9934929.755 | 45027268.35 | 19468729.43 | 27768802.53 | 43834418.74 | 10179756.24 | 10194224.42 |
| L-Cystine | 240.02249 | 1.179 | 39921149.02 | 66570667.26 | 37873085.79 | 77223875.42 | 74220232.72 | 14784856.25 | 36030338.54 | 18096865.02 | 9572652.191 |
| Eleutheroside B | 372.14086 | 7.148 | 32402720.18 | 15918835.32 | 9094470.578 | 4379696.154 | 2144485.886 | 10831434.08 | 8201768.885 | 7986210.625 | 22438480.51 |
| Dehydroepiandrosterone (DHEA) | 270.19682 | 7.418 | 18583693.54 | 15764889.03 | 23954044.85 | 9653988.275 | 19353506.61 | 19786377.59 | 12618733.38 | 8521191.934 | 951677.9983 |
| N-Sinapoylputrescine | 294.15672 | 5.063 | 70029265.87 | 60819253.08 | 40229562.93 | 6902456.252 | 11357712.91 | 12478866.12 | 107077697.4 | 56331950 | 46072658.34 |
| Noroxymorphone | 574.23966 | 8.676 | 19365992.91 | 31672595.61 | 18461380.52 | 3902488.192 | 7288323.605 | 6054805.221 | 4055998.99 | 3762450.883 | 2886609.235 |
| 5α-Dihydrotestosterone | 290.22281 | 7.964 | 22709130.17 | 17857498.26 | 18857041.29 | 135668461.3 | 22524997.15 | 211473502.7 | 57877551.49 | 8130862.454 | 9608206.927 |
| Schizandrol B | 416.18553 | 8.91 | 28221699.66 | 15895058.73 | 19745653.86 | 32918718.66 | 46178880.93 | 42284869.57 | 34972159.69 | 32534065.64 | 24456619.8 |
| N-lactoyl-phenylalanine | 237.099 | 5.751 | 548707439.8 | 301154341.4 | 397215075.3 | 422618392.7 | 519479131.9 | 1151905178 | 529304000.7 | 248658076 | 508597616.4 |
| 3-Hydroxyhippuric Acid | 195.05245 | 5.186 | 116552850.1 | 111450094.3 | 80254880.19 | 37579788.93 | 50858031 | 49606968.77 | 29131389.64 | 23766049.74 | 37350554.12 |
| Ecgonine | 185.10409 | 3.679 | 16755675.35 | 10816396.81 | 7633145.881 | 51069080.91 | 29365981.03 | 61277131.62 | 51518419.4 | 21145793.57 | 36011808.06 |
| N-Isovaleroylglycine | 159.08883 | 5.295 | 371312974.3 | 608199253.4 | 143355357 | 344364198.8 | 1168574583 | 610082951.1 | 347916855.3 | 51802883.92 | 320559095.4 |
| 3-hydroxyquinuclidine-3-carbonitrile hydrochloride | 152.0943 | 5.314 | 3683538571 | 665371856.2 | 1274875886 | 440168648.7 | 448786799.4 | 306238718.9 | 1570636924 | 176924101 | 1645530471 |
| Spectinomycin | 364.1907 | 7.861 | 10436015.81 | 7711585.939 | 8832947.593 | 16495013.94 | 16111168.27 | 26373903.31 | 8575963.961 | 4130943.162 | 5888219.025 |
| lupinine | 169.1469 | 6.368 | 30196071.85 | 8955483.855 | 8599835.007 | 95891488.41 | 24081695.62 | 28349878.81 | 60332792.53 | 2501525.684 | 22564566.44 |
| gamma-Glutamyltyrosine | 310.1149 | 2.072 | 146801906.1 | 114515167.3 | 74047312.48 | 44280049.67 | 64275068.9 | 102824538.3 | 113342330.3 | 85911861.39 | 94976719.03 |
| N1-(5-methylisoxazol-3-yl)-2-morpholinoacetamide | 247.09215 | 4.61 | 43712051.45 | 32173524.55 | 26736659.99 | 45335987.66 | 12647801.31 | 25136627.44 | 43877147.91 | 29612103.79 | 46788861.85 |
| Heptadecanoic Acid | 292.23668 | 8.16 | 66477779.06 | 24114280.3 | 49498263.84 | 174224130.4 | 17326932.27 | 233461874.2 | 2582302.067 | 1746278.385 | 17681553.77 |
| [5-(2-thienyl)-3-isoxazolyl]methanol | 181.01902 | 5.628 | 15807984.04 | 28026477.93 | 11814045.63 | 18030548.41 | 11382672.54 | 30182496 | 12791216.48 | 11173376.39 | 14644523.05 |
| Boc-beta-cyano-L-alanine | 214.09465 | 4.761 | 1057280614 | 650889087.1 | 540156559.4 | 1139480645 | 1768605570 | 1480737483 | 834981135.9 | 213350255 | 335846882.6 |
| Riboflavin | 376.13661 | 5.336 | 90492480.75 | 128444593.6 | 67346059.86 | 54643814.98 | 78886152.33 | 74015798.55 | 56866300.71 | 48607421.71 | 53577901.17 |
| Ergocalciferol | 378.32228 | 6.067 | 26899306.28 | 176657955.1 | 4640454.585 | 60167130.16 | 830167.491 | 4245893.445 | 1065642.049 | 16228790.5 | 11909648.34 |
| Guanidineacetic acid | 117.05435 | 1.46 | 6422852.831 | 2745587.55 | 176049.2176 | 40672002.51 | 29309611.33 | 50251096.12 | 53714664.38 | 36381977.53 | 47139797.43 |
| Epigoitrin | 129.02442 | 5.155 | 3159909.077 | 3503227.881 | 2356150.442 | 5517069.713 | 5612695.096 | 5081968.637 | 3084260.888 | 2356140.972 | 2176595.971 |
| Artesunate | 384.17846 | 7.949 | 1412373.814 | 913584.2765 | 1026601.264 | 1640761.087 | 3035734.906 | 1430999.228 | 1585643.109 | 331756.8698 | 1037412.224 |
| N-Acetylhistamine | 153.08932 | 1.057 | 47735232.1 | 46161453.13 | 25794888 | 64019405.34 | 39977680.31 | 32081706.93 | 49454075.92 | 21140539.12 | 24797534.94 |
| α-Lactose | 359.14082 | 1.365 | 148846870.4 | 140383745 | 197224919.3 | 114509978 | 303239717.3 | 122326551.6 | 124049005.6 | 134013100.3 | 73829312.91 |
| Guanosine | 283.09034 | 2.989 | 153255664.9 | 281811795.3 | 106844258.7 | 56078289.71 | 101269530.9 | 119817019.2 | 164098897.1 | 125188106.4 | 152252165.9 |
| C-hexosyl-apigenin O-feruloylhexoside | 770.20234 | 5.368 | 4651582.29 | 8141065.643 | 4308199.396 | 7024059.088 | 3584305.748 | 10312471.13 | 4510258.049 | 5763331.23 | 5617729.808 |
| 3-Hydroxy-glabrol | 408.19443 | 9.266 | 4047197.055 | 7475334.491 | 50645127.28 | 22005200.59 | 8064940.616 | 29023405.04 | 23817919.65 | 36452022.04 | 48046097.35 |
| Sphingosine (d18:1) | 299.28108 | 6.368 | 135157525.7 | 43050238.72 | 152181141.9 | 17715134.91 | 25970212.4 | 5344248.556 | 49517270.85 | 96169375.94 | 91916791.79 |
| C-pentosyl-luteolin-C-hexoside | 580.14037 | 5.241 | 16022205.94 | 10425173.16 | 13410343.14 | 18080053.05 | 11925115.87 | 23309208.72 | 9079978.888 | 6552201.08 | 9653897.459 |
| Maltitol | 344.13021 | 1.316 | 31375857.62 | 25511699.87 | 21981029.2 | 55855029.66 | 32972597.63 | 43850217.35 | 32658905.84 | 37718347.05 | 30129437.27 |

Table S3

. Differential metabolites of yellow *Daqu* and black *Daqu*

| Name | log2FC | Pvalue | VIP | Regulated |
| --- | --- | --- | --- | --- |
| Lysope 14:0 | -9.136417722 | 0.000590632 | 1.626708699 | down |
| octadec-9-ynoic acid | -8.262712342 | 7.16E-06 | 1.628604562 | down |
| 2-Hydroxyphenylalanine | -7.044420191 | 0.041091255 | 1.492580686 | down |
| Diosgenin | -6.035069117 | 0.029036043 | 1.244183295 | down |
| Corticosterone | -5.492905581 | 0.008915335 | 1.481493456 | down |
| Tetramethylpyrazine | -4.456223238 | 0.001359514 | 1.416057306 | down |
| 6-Methylquinoline | -4.201743187 | 0.046276659 | 1.279259064 | down |
| LPE 15:0 | -3.703677099 | 0.000897014 | 1.297554622 | down |
| DL-Tryptophan | -3.249219131 | 0.001311762 | 1.412690848 | down |
| Prostaglandin J2 | -2.916741467 | 0.041679602 | 1.309866014 | down |
| Sinapinic acid | -2.537981359 | 0.000148207 | 1.639536193 | down |
| Pyridoxal | -2.466373757 | 0.025095025 | 1.19023818 | down |
| Sorbic acid | -2.17571195 | 0.0144923 | 1.391904042 | down |
| α-Linolenoyl ethanolamide | -2.165359409 | 0.001369473 | 1.464344328 | down |
| Diosbulbin B | -2.122760053 | 0.033753777 | 1.630334487 | down |
| Ecgonine | -2.009102717 | 0.011631705 | 1.422561783 | down |
| Indole-3-lactic acid | -1.972142893 | 0.005526983 | 1.369886289 | down |
| (+/-)12(13)-DiHOME | -1.912241963 | 0.000209762 | 1.319620873 | down |
| Betaine | -1.748807074 | 0.004576726 | 1.60809064 | down |
| N-Acetyl-L-leucine | -1.741619819 | 0.03067798 | 1.193714813 | down |
| Xanthine | -1.697889267 | 0.005878551 | 1.121968344 | down |
| PC (14:0e/2:0) | -1.69118651 | 0.03859773 | 1.263773222 | down |
| 3-Phenyllactic acid | -1.60325752 | 0.047092239 | 1.142333411 | down |
| Lithocholic Acid | -1.568145666 | 0.040062944 | 1.063719409 | down |
| N-Acetyl-D-alloisoleucine | -1.551621575 | 0.033979926 | 1.262528084 | down |
| Glutaconic acid | -1.324348293 | 0.047562106 | 1.027105647 | down |
| N-Acetylalanine | -1.310012205 | 0.045932158 | 1.252319037 | down |
| Corchorifatty acid F | -1.295389594 | 0.010263911 | 1.369661092 | down |
| Azelaic acid | -1.289913747 | 0.007981318 | 1.221161887 | down |
| Indole | -1.236436958 | 0.048800276 | 1.340866801 | down |
| Hypoxanthine | -1.193456788 | 0.009425664 | 1.131031633 | down |
| Linoleoyl ethanolamide | -1.163098446 | 0.019550541 | 1.236193215 | down |
| D-Saccharic acid | -1.077364221 | 0.00852487 | 1.330232116 | down |
| N-Acetylornithine | -1.05730016 | 0.03342246 | 1.140350339 | down |
| Pantothenic acid | -1.01154287 | 0.032643312 | 1.033074922 | down |
| N-Oleoyl Glycine | -0.943976941 | 0.003737315 | 1.552948337 | down |
| Schizandrol B | -0.92652112 | 0.037534185 | 1.428995384 | down |
| Uracil | -0.911129522 | 0.047496012 | 1.0513785 | down |
| Epigoitrin | -0.845953002 | 0.030609595 | 1.185998475 | down |
| C-pentosyl-apeignin O-feruloylhexoside | -0.835152187 | 0.003116636 | 1.25619184 | down |
| Trigonelline | -0.812589786 | 0.024486762 | 1.37885541 | down |
| Vincamine | -0.255544255 | 0.041934772 | 1.005893207 | down |
| Coenzyme Q2 | 0.673126952 | 0.011684246 | 1.477968124 | up |
| MAG (18:3) | 1.014304609 | 0.034923922 | 1.252255354 | up |
| 9-HpOTrE | 1.117445194 | 0.032621315 | 1.310930925 | up |
| 3-Hydroxyhippuric Acid | 1.159001089 | 0.006984038 | 1.054221481 | up |
| N-(4-butyl-2-methylphenyl)-N'-[4-(4-methylpiperazino)phenyl]urea | 1.202690003 | 0.016852855 | 1.321247284 | up |
| trans-Petroselinic Acid | 1.259121248 | 0.039364521 | 1.528269893 | up |
| 13(S)-HOTrE | 1.336477339 | 0.017288349 | 1.580862357 | up |
| 1-Stearoylglycerol | 1.542582699 | 0.040210593 | 1.356671724 | up |
| L-Alanyl-L-Lysine | 1.773442501 | 0.034826292 | 1.437766154 | up |
| (±)9-HpODE | 1.843649679 | 0.004291847 | 1.216694013 | up |
| Taxifolin | 1.953366292 | 0.043910798 | 1.576855181 | up |
| Noroxymorphone | 2.010782588 | 0.005362223 | 1.197708828 | up |
| 3-methyl-5-oxo-5-(4-toluidino)pentanoic acid | 2.292807393 | 0.045005204 | 1.39691046 | up |
| Linoleic acid | 2.337642322 | 0.01156469 | 1.338908309 | up |
| Monoolein | 2.35508995 | 0.021207973 | 1.349411206 | up |
| N-Sinapoylputrescine | 2.476511144 | 0.002337105 | 1.349866432 | up |
| Sphingosine (d18:1) | 2.75244047 | 0.03595468 | 1.345291458 | up |
| DL-Panthenol | 2.816025982 | 0.01148804 | 1.315443816 | up |
| N2-Methylguanosine | 3.655596908 | 2.19E-05 | 1.534311707 | up |
| 3-[4-methyl-1-(2-methylpropanoyl)-3-oxocyclohexyl]butanoic acid | 3.802663725 | 0.015859206 | 1.588727802 | up |
| 6-Benzyladenine | 4.704871441 | 0.006431328 | 1.57451064 | up |
| Acetophenone | 4.923779735 | 0.016513819 | 1.623794067 | up |
| (±)9(10)-DiHOME | 7.238196331 | 0.000289101 | 1.622116215 | up |
| Oleoyl ethylamide | 8.761329038 | 0.003035037 | 1.498375344 | up |

Table S4. Differential metabolites of yellow *Daqu* and white *Daqu*

| Name | log2FC | Pvalue | VIP | Regulated |
| --- | --- | --- | --- | --- |
| Methionine sulfoxide | -9.126017914 | 0.000849602 | 1.786604879 | down |
| Lysope 14:0 | -6.397738133 | 0.002055061 | 1.616313127 | down |
| Corticosterone | -6.036222201 | 0.004769414 | 2.335298461 | down |
| N-p-Coumaroyl putrescine | -4.568393633 | 0.000291487 | 2.264826294 | down |
| 4-oxododecanedioic acid | -4.566276237 | 0.001120341 | 2.391522395 | down |
| N-Acetylsphingosine | -4.199115699 | 0.028031306 | 2.195805013 | down |
| Lathyrol | -4.050641484 | 0.005945904 | 2.511095315 | down |
| 6-Methylquinoline | -3.530346052 | 0.034136624 | 1.818083018 | down |
| δ-Gluconic acid δ-lactone | -2.66247231 | 0.012835029 | 1.792915864 | down |
| Kinetin | -2.368553067 | 0.007581822 | 1.57832225 | down |
| Ecgonine | -1.626172467 | 0.032524911 | 1.60056671 | down |
| Betaine | -1.046308482 | 0.023369617 | 1.364327544 | down |
| Ala-trp | -1.007480031 | 0.008564921 | 1.134012656 | down |
| Tropine | -0.896271548 | 0.018377548 | 1.090325889 | down |
| Guanine | -0.871456149 | 0.036597928 | 1.070615937 | down |
| 6-Biopterin | 0.587239107 | 0.035560461 | 1.327103766 | up |
| Kinsenoside | 0.629681525 | 0.013589198 | 1.144284273 | up |
| Senkyunolide A | 0.682513269 | 0.03759181 | 1.182735458 | up |
| DL-Carnitine | 0.723357308 | 0.024016883 | 1.351856795 | up |
| N-Acetylneuraminic acid | 0.952400944 | 0.030398389 | 1.429760424 | up |
| 13(S)-HOTrE | 0.99186866 | 0.030696853 | 1.62097604 | up |
| N-[(-)-Jasmonoyl]-(L)-Isoleucine | 0.995531775 | 0.012990585 | 1.180956402 | up |
| Muramic acid | 1.688628962 | 0.006845234 | 1.740067681 | up |
| 3-Hydroxyhippuric Acid | 1.772170754 | 0.002274958 | 2.260918853 | up |
| D-α-Hydroxyglutaric acid | 1.817677855 | 0.031425028 | 1.86231962 | up |
| Levodopa | 2.106576835 | 0.00245291 | 2.184388503 | up |
| Sedanolide | 2.422195158 | 0.000362952 | 2.075365458 | up |
| D-glutamine | 2.461011553 | 0.01296019 | 1.301697593 | up |
| Noroxymorphone | 2.698719574 | 0.001907007 | 2.224298702 | up |
| 1-Linoleoyl-Rac-Glycerol | 2.733524044 | 0.024303397 | 1.868178429 | up |
| Pipecolic acid | 2.789923653 | 0.001367268 | 1.962230798 | up |
| N1-(2-amino-2-oxoethyl)-2-(isopropylthio)acetamide | 2.916214164 | 0.008200605 | 2.075320166 | up |
| 8-iso Prostaglandin A2 | 3.782288663 | 0.001356279 | 2.533828154 | up |
